# *Helicobacter pylori* targets mitochondrial import and components of mitochondrial DNA replication machinery through an alternative VacA-dependent and a VacA-independent mechanisms

**DOI:** 10.1038/s41598-017-15567-3

**Published:** 2017-11-21

**Authors:** Laurent Chatre, Julien Fernandes, Valérie Michel, Laurence Fiette, Patrick Avé, Giuseppe Arena, Utkarsh Jain, Rainer Haas, Timothy C. Wang, Miria Ricchetti, Eliette Touati

**Affiliations:** 1 Stem Cells and Development, Team Stability of Nuclear and Mitochondrial DNA, Institut Pasteur, 25-28 Rue du Dr. Roux, Paris, France; 2 CNRS UMR3738, Paris, France; 3 Unit of Helicobacter Pathogenesis, Team Genotoxicity, Infection and Cancer, Institut Pasteur, 25-28 Rue du Dr. Roux, Paris, France; 4 CNRS ERL3526, Paris, France; 5 Unit of Human Pathology and Animal Models, Institut Pasteur, 25-28 Rue du Dr. Roux, Paris, France; 6 Paris Descartes University, PRES Sorbonne-Paris-Cité, Paris, France; 7 IRCM (Institut de Recherche en Cancérologie de Montpellier), Université de Montpellier, 34298 Montpellier, France; 8 INSERM U1194, Montpellier, France; 9 Max von Pettenkofer-Institute for Hygiene and Medical Microbiology, Ludwig-Maximilians-University, Pettenkoferstraße 9a, D-80336, Munich, Germany; 10 German Center for Infection Research [DZIF], LMU, Munich, Germany; 11 Division of Digestive and Liver Diseases, College of Physicians and Surgeons, Columbia University, New York, USA; 12 Present Address: UTechS PBI-CiTech, Institut Pasteur, Paris, 75015 France; 13 Present Address: Amity Institute of Nanotechnology, Amity University, Sector 125, Noida, Uttar Pradesh 201313 India

## Abstract

Targeting mitochondria is a powerful strategy for pathogens to subvert cell physiology and establish infection. *Helicobacter pylori* is a bacterial pathogen associated with gastric cancer development that is known to target mitochondria directly and exclusively through its pro-apoptotic and vacuolating cytotoxin VacA. By *in vitro* infection of gastric epithelial cells with wild-type and VacA-deficient *H*. *pylori* strains, treatment of cells with purified VacA proteins and infection of a mouse model, we show that *H*. *pylori* deregulates mitochondria by two novel mechanisms, both rather associated with host cell survival. First, early upon infection VacA induces transient increase of mitochondrial translocases and a dramatic accumulation of the mitochondrial DNA replication and maintenance factors POLG and TFAM. These events occur when VacA is not detected intracellularly, therefore do not require the direct interaction of the cytotoxin with the organelle, and are independent of the toxin vacuolating activity. *In vivo*, these alterations coincide with the evolution of gastric lesions towards severity. Second, *H*. *pylori* also induces VacA-independent alteration of mitochondrial replication and import components, suggesting the involvement of additional *H*. *pylori* activities in mitochondria-mediated effects. These data unveil two novel mitochondrial effectors in *H*. *pylori*-host interaction with links on gastric pathogenesis.

## Introduction

Mitochondria are essential organelles not only responsible for energy production but also involved in apoptosis, calcium homeostasis, lipids and amino acids metabolism. Targeting mitochondria has emerged as a key strategy for bacteria to hijack host cells physiology and promote infection^1,2^. However, the underlying mechanisms and their relevance to disease remain to a great extent unresolved. Virulence factors of both intracellular and extracellular bacteria are secreted in the host cells and may interact with mitochondria, leading to modulation of mitochondrial function and ultimately promoting pathogenesis^3–5^. Mitochondria have also been reported as modulators of cellular antibacterial immunity and inflammatory response^6^. *Helicobacter pylori* is a human gastric pathogen and a major risk factor for gastric cancer^7,8^. *H*. *pylori* damages gastric cells introducing genetic instability and mitochondrial dysfunction, which largely contribute to the infection-associated pathogenicity^9–12^. To date, the pro-apoptotic cytotoxin VacA is the only known *H*. *pylori* protein which targets mitochondria, and is a major virulence factor^13^. In gastric epithelial cells, VacA localizes to endosomal compartments and reaches the mitochondrial inner membrane where it forms anion-conductive channels^14–16^. VacA decreases mitochondrial membrane potential leading to reduced ATP production and cytochrome c release^13^. VacA channel activity disrupts the morphological dynamic of mitochondria through the recruitment and activation of dynamin-related protein 1, an essential factor of mitochondria fission, resulting in BAX/BAK activation and host cell death^17^. VacA is also an efficient inducer of autophagy^18^.

Mitochondria carry multiple copies of their own genome organized into nucleoids, which include the nuclear-encoded DNA polymerase γ (POLG) and transcription factor A (TFAM)^19^. TFAM also helps maintaining mitochondrial DNA (mtDNA) integrity. We previously reported that *H*. *pylori* induces mtDNA mutations in gastric epithelial cells, also observed in gastritis patients, indicating an early occurrence of mtDNA instability during disease progression^20^. *H*. *pylori* also impairs mtDNA repair pathways^21^.

To date, the extent of mitochondrial dysfunctions during *H*. *pylori* infection and their consequences for initiation of gastric pathogenesis remain poorly understood. In the present study, we identify novel mitochondrial targets modulated by *H*. *pylori* during its interaction with the host cells. We show that *H*. *pylori* promotes an early and transitory alteration of mitochondrial import translocases, TOM22 and TIM23, and a dramatic up-regulation of POLG and TFAM. These effects are not exclusively VacA-dependent, and are compatible with host cell survival. Compatible mitochondrial alterations, including the deregulation of mtDNA replication and transcription factors and the depletion of mtDNA during chronic infection, also occur during the progressive evolution of gastric inflammatory lesions toward severity in mice, pointing to their potential role in infection-associated pathogenicity.

## Results

### *H. pylori* increases the mitochondrial mass, deregulates mitochondrial translocases, and decreases mtDNA content in INS-GAS mice

The consequences of *H*. *pylori* on mitochondria were first analysed in INS-GAS mice in which the infection exacerbates the severity of gastric lesions^22,23^. Mice were infected for 6 and 12 months with the *H*. *pylori* strain SS1^24^. As reported^22,23^, infected mice developed inflammatory lesions with higher histological scores for infiltration of inflammatory cells, loss of triangular-shaped parietal cells, and increase of hyperplasia and dysplasia compared to non-infected mice (Supplementary Figure S1A–C). Development of low-grade gastrointestinal intraephithelial neoplasia (GIN) was observed in 30% of mice at 12 months post-infection (pi).

**Figure 1:**
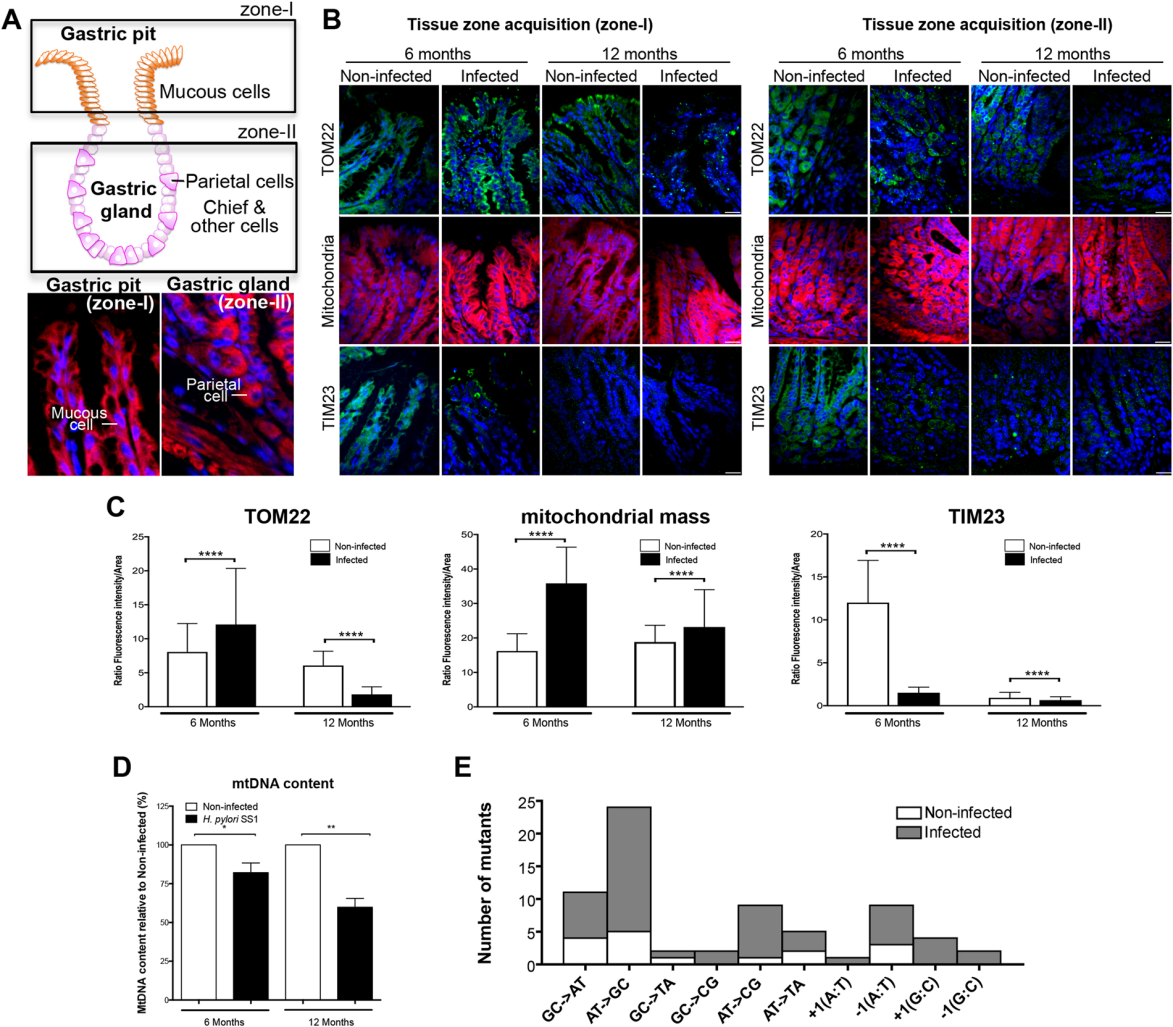
*H*. *pylori* deregulates mitochondrial mass and mitochondrial translocases in INS-GAS mice. (**A**) Structure and cell types in gastric glands. Rectangles indicate the zones (I and II) that have been analysed. The expected cell types in each zone are indicated in the panels below (red, Mitotracker, blue, Hoechst). (**B**) TOM22, TIM23 immunofluorescence and MitoTracker staining on gastric pits (zone I, left panels) and gastric glands (zone II, right panels) on tissue section from 6–12 months *H*. *pylori* SS1-infected INS-GAS mice. (**C**) Quantification of fluorescence intensity (performed on 1600 cells (750 cells in zone I, 750 non-triangular shaped cells and 100 triangular shaped cells, which are less numerous (*i*.*e*. parietal cells, identified with independent staining with anti-intrinsic factor, not shown), in zone II) per condition. Mean ± SD from 3 independent experiments; ****p < 0.0001, Welch’s test, infected (*H*. *pylori* SS1) *vs* non-infected. Optical slices were taken every 200-nm interval along the z-axis covering the whole depth of the tissue, and quantification was performed on 2D images generated from 3D volume rendering (see details in supplementary material). (**D**) Quantification of mtDNA by qPCR in the gene cytochrome c oxidase 1. Mean ± SD from 3 independent experiments; *p < 0.05; **p < 0.01. (**E**) Mutations spectra determined in the mtDNA D-loop region from gastric mucosa of infected and non-infected mice after 6 and 12 months. MtDNA mutations were analysed between nucleotides 41 and 843 in the mtDNA D-loop sequence. Seventy-five percent of *H*. *pylori-*induced mutations were substitution base pairs, half of them correspond to AT- > GC transitions.

The mitochondrial content was assessed in the gastric mucosa (Fig. 1A). MitoTracker Deep Red staining, which labels mitochondria, increased in the gastric tissue upon infection (2.2- and 1.4-fold at 6 and 12 months, respectively, Fig. 1B,C). Immunofluorescence of TOM22, a component of the mitochondrial translocase outer membrane (TOM) complex^25^, which is also indicative of the organelle content^26^, increased at 6 months pi, but decreased at 12 months pi, raising the question whether mitochondrial translocases were affected upon infection. Precursor proteins that must reach the mitochondrial matrix translocate first through the TOM complex then to the translocase inner membrane (TIM) complex, which includes TIM23^27^. TIM23 signal decreased 7-fold in the gastric tissue 6 months pi, and remained very low after 12 months, as in non-infected mice. Dramatically reduced immunostaining signal did not appear to result from cell apoptosis, which increased to a limited extent in infected mice after 12 months, as demonstrated by cleaved Caspase-3 Western blots (WB) (Supplementary Fig. S2A). Moreover, the gastric tissue displayed increased levels of the canonical NF-κB factor p50, and to some extent of the autophagy marker LC3B (Supplementary Fig. S2B), after 12-month infection, in agreement with the activation of pro-inflammatory signaling during long-term infection in these mice. In these cells, *H*. *pylori* chronic infection was associated with progressive depletion of the mtDNA (Fig. 1D) and a 2.8-fold increase in mutation frequency in the D-loop (hypervariable region), probably as a result of mtDNA damage. A mutation frequency of 44.5% (53/119 clones) was observed in *H*. *pylori* infected cells *versus* 16% (16/100) in non-infected mice. Interestingly, a specific mutation spectrum mainly composed of AT- > GC transitions (37%) and frameshift events (25%) was observed in the gastric mucosa of infected mice (Fig. 1E).

In this context, which is compatible with altered mitochondrial function, *H*. *pylori* may trigger oxidative stress, as suggested by decrease of the expression of antioxidant superoxide dismutases (*SOD1*, *SOD2*) and catalase (at 6 months pi) in infected mice (Supplementary Fig. S1D).

### Induction of POLG and TFAM in *H. pylori* chronically infected INS-GAS mice

**Figure 2:**
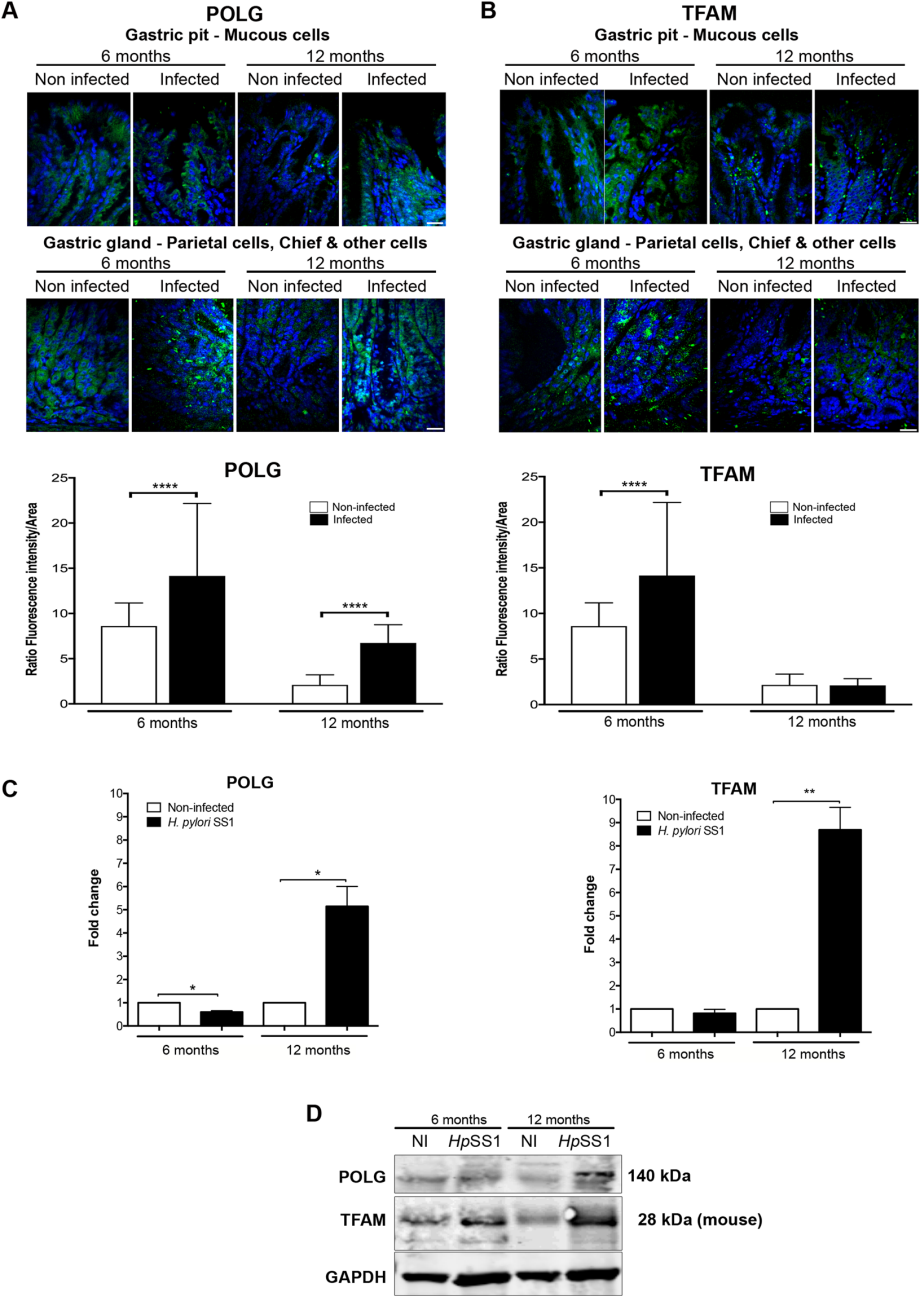
*H*. *pylori* induces POLG and TFAM in INS-GAS mice. (**A**) POLG and (**B**) TFAM immunofluorescence intensity (upper panels) and quantification (lower panels) on gastric tissue section of *H*. *pylori* SS1-infected INS-GAS mice. 1600 cells were assessed per condition, as described in Fig. 1C. Mean ± SD from 3 independent experiments; ****p < 0.0001, Welch’s test, infected (*H*. *pylori* SS1) *vs* non-infected. (**C**) Expression of genes coding for *PolG* and *Tfam* quantified by RT-qPCR from RNA isolated from the gastric mucosa of infected and non-infected mice at each time-point. Values represent the mean ± SD of three independent measurements for each group of mice. Infected mice compared to non-infected; *p < 0.05; **p < 0.01. (**D**) Western blot on total gastric proteins. The 28 kDa isoform of TFAM (mouse) is prevalent. Samples were derived from the same experiment and the gel and blots were processed in parallel. The blot was then sliced in three horizontal sections to allow multiple immunolabelings with the same membrane. Sections were alternatively labeled with anti-POLG, anti-TFAM, or anti-GAPDH. The corresponding uncropped gel/blots are shown in Supplementary Figure S7B.

Alteration of translocases may affect the import of key proteins into mitochondria and thereby their intracellular levels. In sections of the gastric mucosa, immunofluorescence of the mtDNA polymerase γ (POLG, catalytic subunit) increased 2- and 3-fold six and 12 months pi, respectively (Fig. 2A), the last point in agreement with WB of gastric protein extracts (Fig. 2D). Immunofluorescence of the mtDNA maintenance factor TFAM increased by 20% six months pi, whereas after 12 months was as low as in non-infected mice (Fig. 2B). In protein extracts of the gastric tissue, WB revealed an increase of TFAM after 12 months (Fig. 2D) correlated with mRNA levels (Fig. 2C). Although these tests target the same tissue, differences in gastric sections *versus* total gastric tissue, and the occurrence of epitopes in the natural conformation in immunofluorescence but not necessarily in WB, may explain differences observed with these two approaches. Thus, POLG, and at a minor extent TFAM, were enriched in the gastric mucosa of infected mice, at least 12 months pi.

### *H. pylori*-induced early and transient increase of mitochondrial translocases

**Figure 3:**
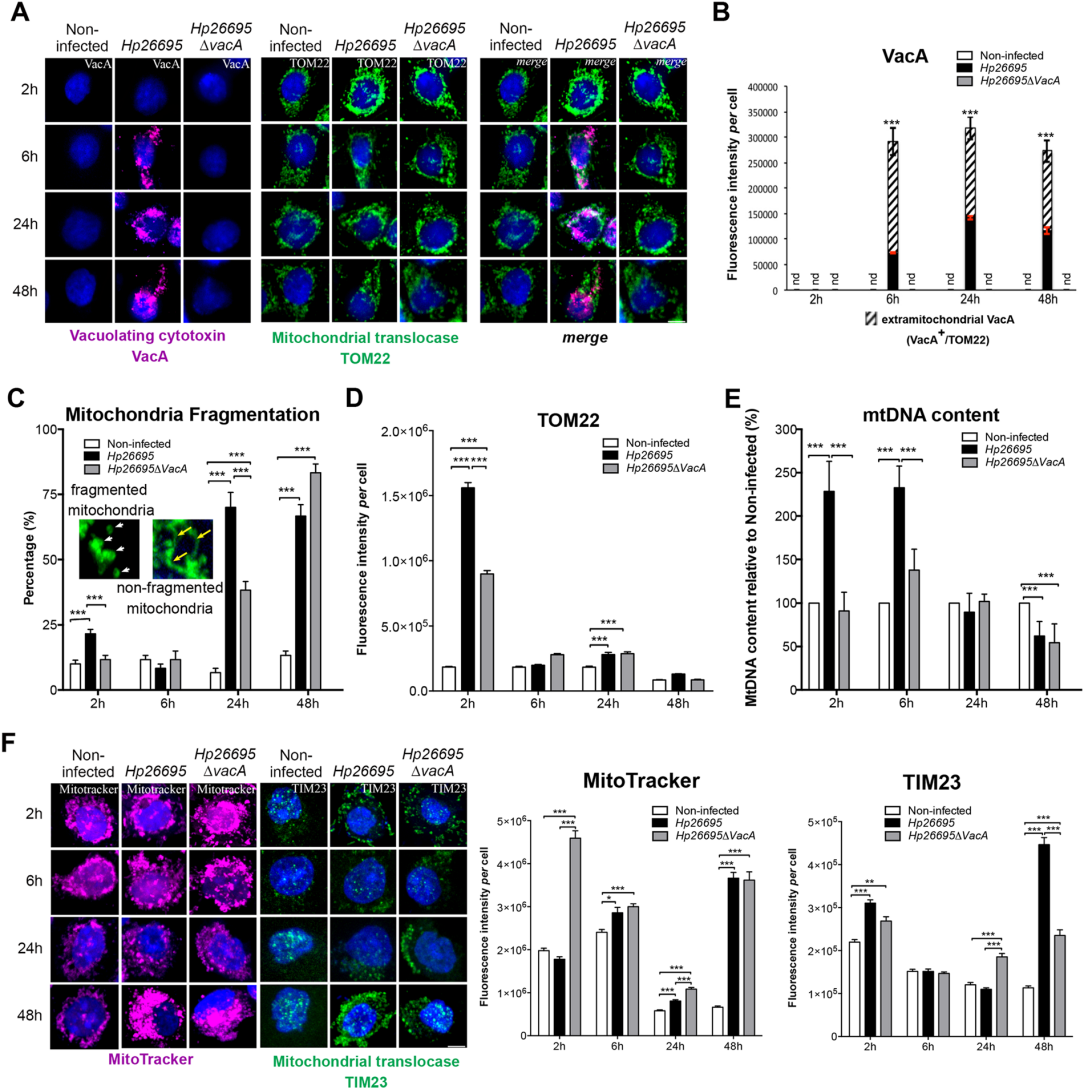
Transient alteration of mitochondrial translocases early upon *H*. *pylori* infection. AGS cells co-cultured with *H*. *pylori* 26695 and 26695∆*vacA*. (**A**) 3D-reconstructed immunofluorescence of cells labelled with VacA (purple), TOM22 (green), and counterstained with Hoechst (nuclei, blue). (**B**) Quantification of VacA immunofluorescence. Extramitochondrial VacA (VacA^+^/TOM22^-^, hatched) and intramitochondrial VacA (VacA^+^/TOM22^+^, black); error bars in red; no detection of VacA (“nd” for “not detected”) in non-infected cells and cells infected with the *H*. *pylori* 26695∆*vacA*. For infection with *Hp*26695, intramitochondrial VacA (in black in the histogram) was also tested at different time points with one-way ANOVA and Dunn’s multiple comparisons test, in both cases resulting in p < 0.0001 (**** in red); differences in total VacA in these conditions were not significant. (**C**) Percentage of cells displaying mitochondrial fragmentation (n = 60 cells/condition, from 3 independent experiments, 2-tailed Fisher’s exact test). Insets (5x magnification) illustrate fragmented (white arrowheads) and non-fragmented (yellow arrows) mitochondria. (**D**) Quantification of TOM22 immunofluorescence intensity/cell. (**E**) MtDNA content quantified by qPCR (mean ± SD). (**F**) 3D-reconstructed fluorescence of cells labelled with MitoTracker Deep Red (purple), TIM23 immunostaining (green), and nuclei (Hoechst, blue) and fluorescence quantification. In panels D and F, mean ± SD, n = 30 cells/conditions from 3 independent experiments, Welch’s test. *p < 0.05; **p < 0.01; ***p < 0.001; ****p < 0.0001. Scale bar: 10 µm.

*H*. *pylori*-mediated deregulation of mitochondrial components and the role of the VacA cytotoxin were then further investigated in AGS gastric epithelial cells infected for 2 h–48 h with the *H*. *pylori* strain 26695 and the isogenic ∆*vacA* mutant (constructed as described in the supplementary informations). In *H*. *pylori* 26695-infected cells, VacA was detected intracellularly from 6 h (Fig. 3A,B). Co-immunolabeling of VacA and TOM22 suggested that most mitochondria (78% and 87% at 6 h and 48 h, respectively) were targeted by VacA (TOM22^+^/VacA^+^, merge in Fig. 3A), despite the cytotoxin remained largely extramitochondrial (Fig. 3B).

Infection with a VacA-proficient strain has been previously associated with apoptosis-related mitochondrial fragmentation 8 h pi^17^. Upon infection, we observed an increase of mitochondrial fragmentation at later time points (up to 70 ± 10% and 83.3 ± 5.8% with the *Hp*26695 and *Hp*26695*∆VacA* strain, respectively, at 48 h pi) compared to 13.33 ± 2.9% in non-infected cells (Fig. 3C). However, despite mitochondrial fragmentation, apoptosis in infected cells was very low as indicated by ≈5% Annexin-V staining at both late time-points (Supplementary Figure S3B).  In addition, we did not detect cleaved Caspase-3 and the downstream factor cleaved Caspase-6, and did not observe significant increase of cleaved PARP-1, a further substrate of caspases^28^ (Supplementary Figure S3D). Moreover, cell cycle progression was maintained. Indeed, although we noticed an increase of the fraction of cells in G0/G1 at 24 h (*p* = *0*.0003), compatibly with a recent report^29^, infected cells proliferated as much as non-infected cells at 48 h (Supplementary Figure S3A, C).

Interestingly, a large fraction of cells with mitochondrial fragmentation was also observed at 24 h (38 ± 5.7%) and 48 h (83 ± 5.7%) pi in the absence of VacA. Under these conditions, the autophagy marker LC3B was specifically up-regulated 24 h pi with the *H*. *pylori* strain 26695 and to a minor extent with the 26695∆*vacA* strain (Supplementary Figure S3E). At 24 h the 26695 strain also triggered expression of the NF-κB factor p50 (Supplementary Figure S3F). Thus, *H*. *pylori* infection of AGS cells globally displays up-regulation of autophagy and pro-inflammatory markers (24 h and 48 h), which were observed in the gastric mucosa of long-term (12-month) infected mice (see above Supplementary Fig. S2B). Conversely, differently from infected mice, infected cells did not display increased apoptosis markers upon short time infection.

The mitochondrial content, assessed by MitoTracker Deep Red signal, was unchanged in *H*. *pylori* 26695-infected cells 2 h pi, but increased by 2.5-fold in 26695∆*vacA-*infected cells, compared to non-infected controls (Fig. 3F). After returning to almost control levels, the mitochondrial mass was induced again 48 h pi, in a VacA-independent manner. Conversely, TOM22 immunosignal^26,30^ transiently increased by 8-fold in 2 h *H*. *pylori* 26695-infected cells, when VacA was extracellular (or too low in concentration to be detected inside cells), and by 5-fold in 26695∆*vacA-*infected cells (Fig. 3D). These data suggested that the content of mitochondrial translocases, and their ratio *versus* mitochondrial mass, were altered upon *H*. *pylori* infection, as it was the case during *in vivo* infection (see above, Fig. 1). Normalization of translocases to MitoTracker (mitochondrial mass) confirmed that the TOM22 signal was 9-fold and TIM23 1.6-fold enriched 2 h pi with *H*. *pylori* 26695 (Fig. 3D, F; Supplementary Table S2). Thus, *H*. *pylori* infection resulted in a bi-phasic alteration of mitochondrial translocases and mitochondrial mass: enrichment at 2 h pi and depletion at 48 h pi (TOM22), with an intermediary period characterized by restoration to control values. Remarkably, the largest alterations of translocase factors, especially TOM22, occurred early pi when VacA was not detected intracellularly.

Mitochondrial alterations at 2 h pi were associated with a VacA-dependent increase of the mtDNA content that persisted at 6 h (Fig. 3E). MtDNA was then depleted at 48 h pi in a VacA-independent manner, as previously described^21^, and in agreement with depletion of mtDNA during chronic infection in mice (see above, Fig. 1). Depletion of mtDNA in spite of maintained mitochondrial mass has been described in pathological conditions^26,31–33^.

Thus, *H*. *pylori*-mediated early and transient alteration of mitochondrial translocases and mtDNA content occured when VacA was not detected intracellularly. Remarkably, to a lower extent other activities of *H*. *pylori* besides VacA also seem to affect these mitochondrial factors.

### *H. pylori* and VacA modulate the expression of POLG and TFAM and affect their cytosolic *versus* mitochondrial localization

**Figure 4:**
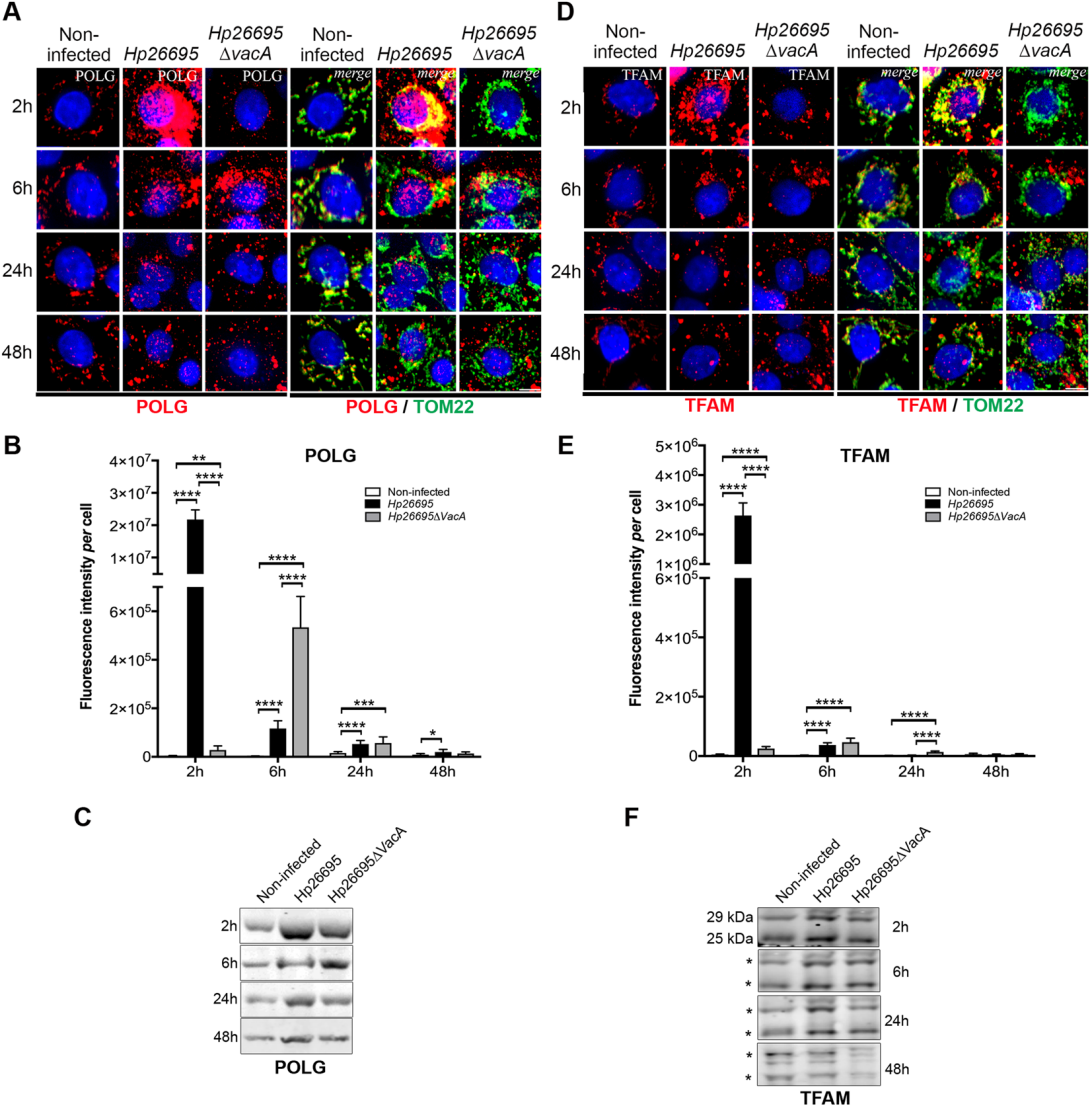
VacA-dependent and VacA-independent increase of POLG and TFAM early upon *H*. *pylori* infection. AGS cells co-cultured with *H*. *pylori* 26695 or 26695∆*vacA*. (**A**) POLG and (**D**) TFAM immunofluorescence (red, left panels); merge with TOM22 immunostaining (green) and nuclei (Hoechst, blue) (right panels). Scale bar: 10µm. (**B**) POLG and (**E**) TFAM quantification of immunofluorescence intensity (n = 30 cells/condition (and n = 50 non-infected cells) from three independent experiments mean ± SD, Welch’s test, **p < 0.01; ***p < 0.001; ****p < 0.0001. WB of (**C**) POLG and (**F**) TFAM (human 29 kDa and 25 kDa isoforms, position indicated with a star). Samples were derived from the same experiment and the gels and blots were processed in parallel (one gel for 2 h and 6 h, one gel for 24 h and 48 h). Each blot was then sliced in horizontal sections to allow multiple immunolabelings with the same membrane. Sections were alternatively labeled with anti-POLG and anti-TFAM (other labelings performed with the remaining portions of the membrane are not shown). The corresponding uncropped gels/blots are shown in Supplementary Figure S7C. SYPRO Ruby blot staining used as loading control (total protein staining is used as alternative to overcome single housekeeping protein variations) is shown in Supplementary Figure S7C (entire gel staining). Data from 3 independent experiments.

We then investigated POLG and TFAM levels in infected cells. Immunofluorescence labeling showed a dramatic increase of POLG in *H*. *pylori* 26695-infected cells after 2 h when VacA was not detected intracellularly (Fig. 4A,B). Later upon infection, POLG signal progressively decreased to control levels, as confirmed by WB (Fig. 4B,C). Importantly, POLG signal was less pronounced in *H*. *pylori* 26695∆*vacA*-infected cells, but was nevertheless 10-fold (2 h) and 5-fold (6 h) higher than controls (Fig. 4A,B). Thus, increase of POLG also occurred independently of VacA but to a lower extent and delayed compared to the wild-type infection, suggesting that *H*. *pylori* activities other than VacA can modulate POLG levels.

Interestingly, *H*. *pylori* affected the subcellular localization of POLG. Figure 4A and Supplementary Table S3A show that in the presence of VacA, most mitochondria displayed POLG signal (TOM22^+^/POLG^+^ labelling), although some POLG was also located outside mitochondria (POLG signal that did not co-localize with TOM22). Conversely, in the absence of VacA, POLG was initially concentrated in a few mitochondria (6.42 ± 0.9% of the signal at 2 h) but later upon infection the situation becomes similar as for the wild-type strain. In non-infected cells, under all conditions, POLG was apparently located in mitochondria (TOM22^+^ regions), as expected.

As for POLG, a dramatic and early accumulation of TFAM was observed in *H*. *pylori* 26695-infected cells after 2 h, which returned to almost normal levels at 48 h pi (Fig. 4D,E). *H*. *pylori* 26695∆*vacA* also induced TFAM at 2 h, but the signal was 100-fold lower than with the 26695 strain. Alterations of TFAM levels were confirmed by WB (Fig. 4F). Upon infection with either strains, most (≥71.3%) TFAM appeared localized in mitochondria (TFAM^+^/TOM22^+^), although in the absence of VacA, TFAM appeared concentrated in a few mitochondria (19.0 ± 1.4% of signal) at 2 h pi (Supplementary Table S3B). Thus, early upon infection and in the absence of VacA, only a small fraction of mitochondria was enriched in POLG and TFAM, despite extramitochondrial POLG and to a minor extent TFAM were observed with either *H*. *pylori* strains.

The transient induction of POLG and TFAM by *H*. *pylori* slightly affected mtDNA initiation of replication and transcription, as revealed by mTRIP^30^ imaging that detects accessible mitochondrial nucleic acids in single cells, and by RT-qPCR of mitochondrial-coded genes (Supplementary Figure S4). Finally, as in chronically infected INS-GAS mice (see above, Supplementary Figure S1D), *H*. *pylori* strongly reduced the antioxidant defence, inferred by decreased expression of SOD1, SOD2, and catalase at 24–48 h pi (Supplementary Figure S5A).

Altogether, *H*. *pylori* efficiently and transiently induced the key mtDNA replication and transcription factors POLG and TFAM, and affected their cytosolic *versus* mitochondrial localization. Early accumulation of these factors occurred when VacA was not detected intracellularly. Our data also suggest the involvement of other *H*. *pylori* activities than VacA in these events.

### Purified VacA is sufficient to alter the levels of mitochondrial translocases and mtDNA

**Figure 5:**
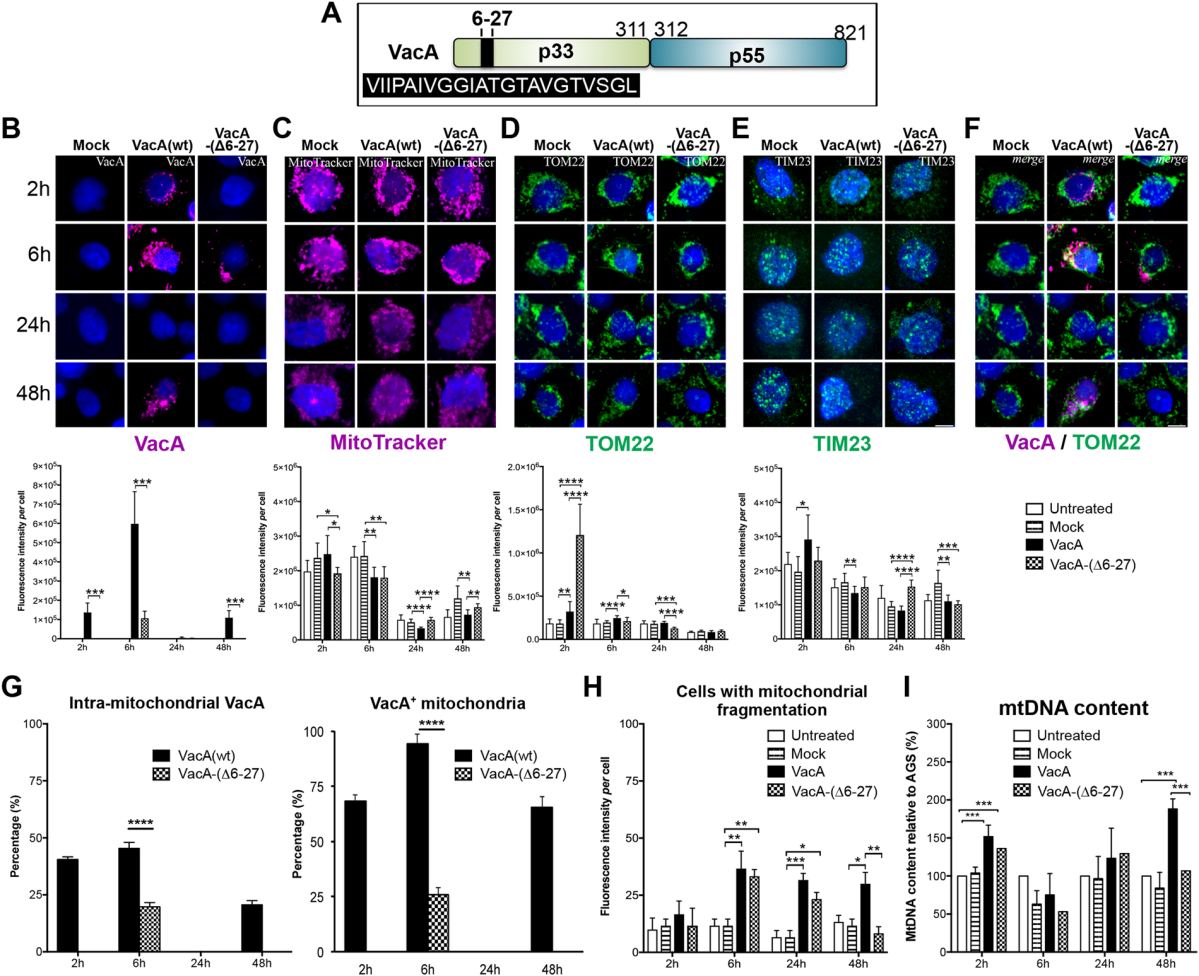
Isolated VacA affects mitochondria and mtDNA content. AGS cells incubated with acid-activated VacA(wt) and VacA-(∆6-27)^35^. (**A**) Schematic representation of VacA domains. The black box indicates the aminoacid composition of the N-terminal hydrophobic region deleted in VacA-(∆6-27). (**B**) VacA (purple), (**C**) mitochondria labelling by MitoTracker (purple), (**D**) TOM22 (green), (**E**) TIM23 (green) immunostaining, (**F**) VacA and TOM22 merge (right panel). Nuclei (Hoechst, blue). Scale bar: 10 µm. Quantification of immunofluorescence intensity below each panel (n = 30 cells/condition from three independent experiments (panels B and D n = 6), mean ± SD, Welch’s test). (**G**) Percentage of intra-mitochondrial VacA(wt) and VacA-(∆6-27) (VacA^+^TOM22^+^/total VacA labelling, left panel) and mitochondria that co-localize with VacA (VacA^+^TOM22^+^/total TOM22 labelling, right panel), n = 6 experiments, Welch’s test. (**H**) Percentage of cells with mitochondria fragmentation (n = 60 cells/condition, from 3 independent experiments, 2-tailed Fisher’s exact test). (**I**) Quantification of mtDNA by qPCR; mean ± SD from n = 3 experiments. Untreated: AGS cells; Mock: AGS cells incubated with the VacA activation buffer as control. Values of untreated cells in panels C-E are the same as in Fig. 3F since these experiments and the relative acquisitions were done at the same time. VacA or VacA-(-∆6-27) *versus* Mock (in panels B and G: VacA *versus* VacA-(∆6-27)); *p < 0.05; **p < 0.01; ***p < 0.001; ****p < 0.0001.

In order to investigate whether the VacA cytotoxin alone deregulates mitochondrial components, cells were incubated with acid-activated VacA(wt), purified as described^34,35^. As control, cells were incubated with proteinVacA-(∆6-27) (Fig. 5A), which is partially deficient in cellular uptake, lacks vacuolating activity and fails to integrate into the inner-mitochondrial membrane^16,34^. VacA(wt) was detected inside cells after 2 h (Fig. 5B), as expected^17^, peaked at 6 h, dropped at 24 h, and was detected again at 48 h, suggesting a second phase of internalization. VacA-(∆6-27) was internalized to a lower extent and slower (by 6 h) than VacA(wt) and without a second internalization by 48 h. VacA(wt) had little effect on the mitochondrial mass (MitoTracker, Fig. 5C), but transiently increased (1.7-fold) TOM22 signal at 2 h. Although not detectable intracellularly, at 2 h VacA-(∆6-27) induced 6.6-fold the TOM22 signal (Fig. 5D) with essentially no effect on TIM23 (Fig. 5E) and mitochondrial mass (Fig. 5C), corroborating the effect of VacA in *H*. *pylori* 26695-infected cells during the 2 first hours of infection, which was also not detected intracellularly (see above Fig. 3D,F).

VacA-(∆6-27) did not reach mitochondria as efficiently as VacA(wt), as expected^16,34^, with only 26.0 ± 4% of VacA-positive mitochondria after 6 h compared to 94.3 ± 2% with VacA(wt) (Fig. 5G). Nevertheless, at 6 h wt and truncated-VacA increased by 2-fold the fraction of cells with mitochondrial fragmentation (Fig. 5H), supporting the observed mitochondrial fragmentation in late *H*. *pylori* 26695-infected cells (see above, Fig. 3C).

The mtDNA content transiently increased after 2 h exposure to VacA(wt) or VacA-(∆6-27), and was induced again at 48 h (Fig. 5I), concomitantly with a second phase of accumulation of VacA(wt) in cells. In summary, isolated VacA induced mitochondrial fragmentation, deregulated mitochondrial translocases and mtDNA content similarly to cytotoxin-mediated action upon *H*. *pylori* 26695 infection.

### Functional VacA and to a minor extent VacA-(∆6-27) increase POLG and TFAM levels

**Figure 6:**
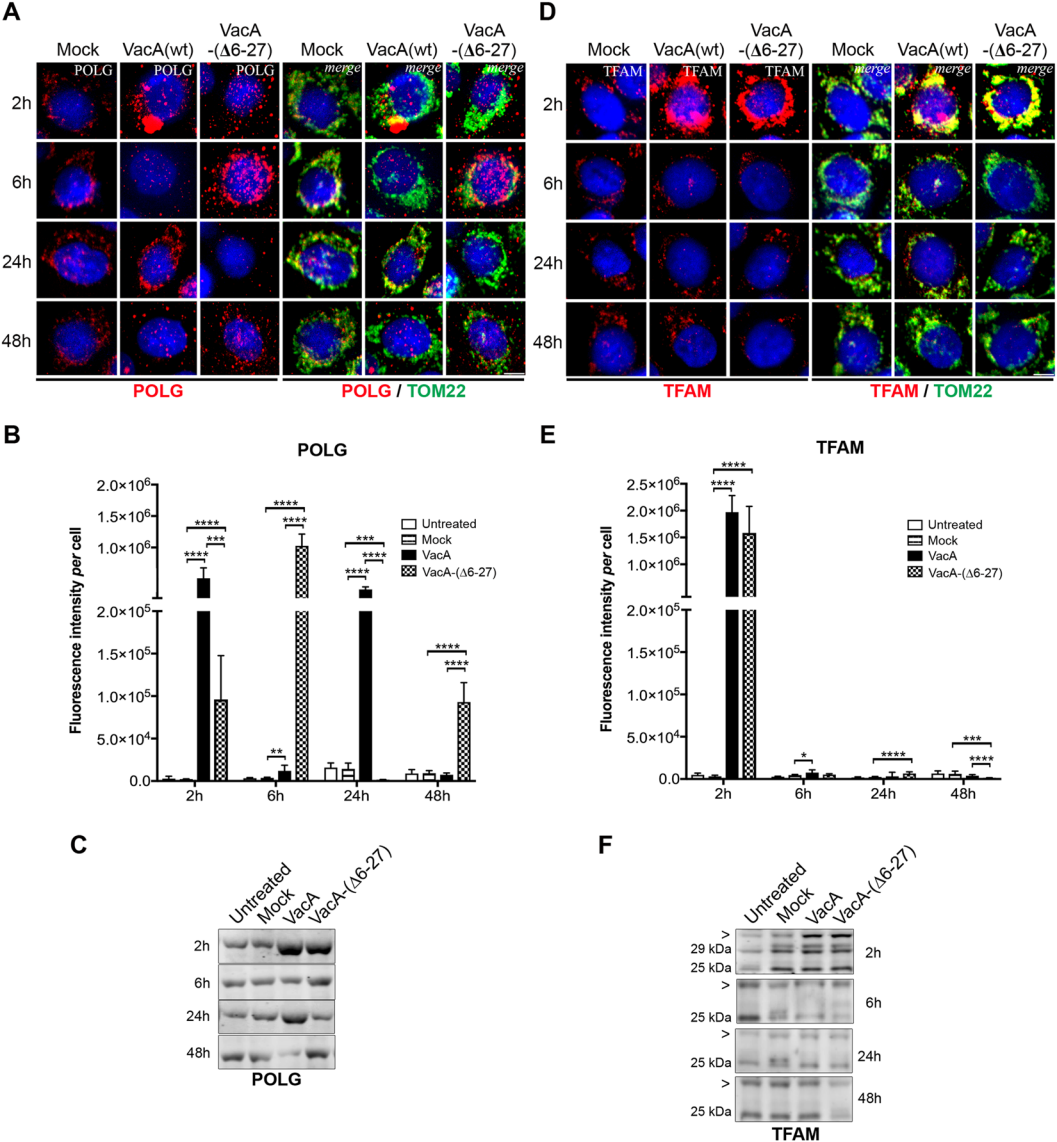
VacA leads to early and transient accumulation of POLG and TFAM. AGS cells incubated with acid-activated VacA(wt) and VacA-(∆6-27)^35^. (**A**) POLG and (**D**) TFAM visualized by confocal immunofluorescence (red; left panels). The right panels show merge with mitochondrial immunostaining staining (TOM22, green). Nuclei (Hoechst, blue). Scale bar: 10 µm. Quantification of POLG (**B**) and TFAM (**E**) immunofluorescence intensity (n = 30 cells/condition (and n = 50 untreated cells) from three independent experiments, mean ± SD, Welch’s test, *p < 0.05; **p < 0.01; ***p < 0.001; ****p < 0.0001, *versus* untreated AGS). Untreated: AGS cells; Mock: AGS cells incubated with the VacA activation buffer as control. WB of (**C**) POLG and (**F**) TFAM. The 29 kDa and 25 kDa isoforms of TFAM, as well as a higher molecular weight band ( > , at ≈ 31-32 kDa) are present in all samples at 2 h, whereas at the other time points the 25 kDa isoform and the larger band are prevalent. Samples were derived from the same experiment and the gels and blots were processed in parallel (one gel for 2 h and 6 h, one gel for 24 h and 48 h). Each blot was then sliced in horizontal sections to allow multiple immunolabelings with the same membrane. Sections were alternatively labeled with anti-POLG and anti-TFAM (other labelings performed with the remaining portions of the membrane are not shown). The corresponding uncropped gels/blots are shown in Supplementary Figure S7D. SYPRO Ruby blot staining used as loading control (total protein staining is used to overcome single housekeeping protein variations) is shown in Supplementary Figure S7D (entire gel staining).

VacA purified protein alone increased POLG immunofluorescence by 183-fold with VacA(wt) and 34-fold with VacA-(∆6-27) after 2 h (Fig. 6A,B). Intracellular VacA-(∆6-27) accumulated with delay, and after 6 h led to an increase of the POLG signal (331-fold). POLG induction was confirmed by WB (Fig. 6C). VacA seems also to affect the ability of POLG to reach or be imported into mitochondria, as only 33% and 1.2% of mitochondria display POLG signal after 2 and 6 h of incubation with VacA(wt), respectively (Supplementary Table S4A).

VacA also induced a transient accumulation of TFAM immunofluorescence by 2 h with 396-fold (VacA(wt)) and 318-fold (VacA-(∆6-27)) increase of the signal compared to untreated cells (Fig. 6D,E). After 2 h incubation with both forms of VacA, essentially all mitochondria appeared to contain TFAM (TOM22^+^/TFAM^+^), although this fraction decreased to 41-45% by 6 h, when VacA was intracellular (Supplementary Table S4B).

Early accumulation of POLG and TFAM in cells treated with VacA(wt) occurred under robust oxidative stress response, as suggested by high expression of SOD1 and catalase, (Supplementary Figure S5B). Differently from *H*. *pylori* 26695-infected cells, the antioxidant response was essentially not reduced by either form of VacA, suggesting the involvement of other *H*. *pylori* activities in this process.

In summary, VacA alone induced POLG and TFAM, independently of the presence of its N-terminal hydrophobic domain. The extracellular presence of VacA was sufficient to trigger this induction as also observed during the first hours of infection *in vitro*.

## Discussion

Targeting mitochondria is a relevant strategy for pathogens to affect host cells, by promoting or, alternatively, circumventing cell death^1,2,36^. Mitochondria participate in many cell signaling events, and their alteration has been linked to acute and chronic inflammation^37^. *H*. *pylori* is known to target mitochondria through its vacuolating cytotoxin VacA^13^. Here we show that *H*. *pylori* affects mitochondria by two additional mechanisms.

First, *H*. *pylori* transiently deregulates mitochondrial translocases, dramatically increases the levels of the mitochondrial DNA polymerase POLG, the transcription/DNA maintenance factor TFAM, and the mtDNA content. These alterations, which are to a great extent VacA-dependent, do not promote apoptosis and are associated with survival of infected cells. These data raise the question of the balance between pro-apoptotic and potentially pro-survival properties of VacA. *H*. *pylori* has been reported to have apparently contradictory effects on the cell cycle and cell survival, and a recent study reported cell cycle arrest upon infection of AGS cells with the 29665 strain^29^, which actually consisted in mild alterations of the cell-cycle dynamics. Compatibly with these findings, we observed a slight delay in cell cycle progression upon infection which, however, did not prevent infected cells to proliferate as much as non-infected cells. We consider these alterations a delay rather than an arrest of the cell cycle. Secondly, part of these alterations is VacA-independent, pointing to additional *H*. *pylori* activities that affect mitochondria.

We also show that mitochondrial translocases, as well as POLG and TFAM are affected *in vivo* upon chronic *H*. *pylori* infection (6–12 months) in INS-GAS mice that develop GIN^22,23^. These data highlight that *H*. *pylori-*mediated mitochondrial effects occur in parallel with the progression of gastric inflammatory lesions towards severity, suggesting the contribution of mitochondrial alterations to *H*. *pylori* pathogenesis.

**Figure 7:**
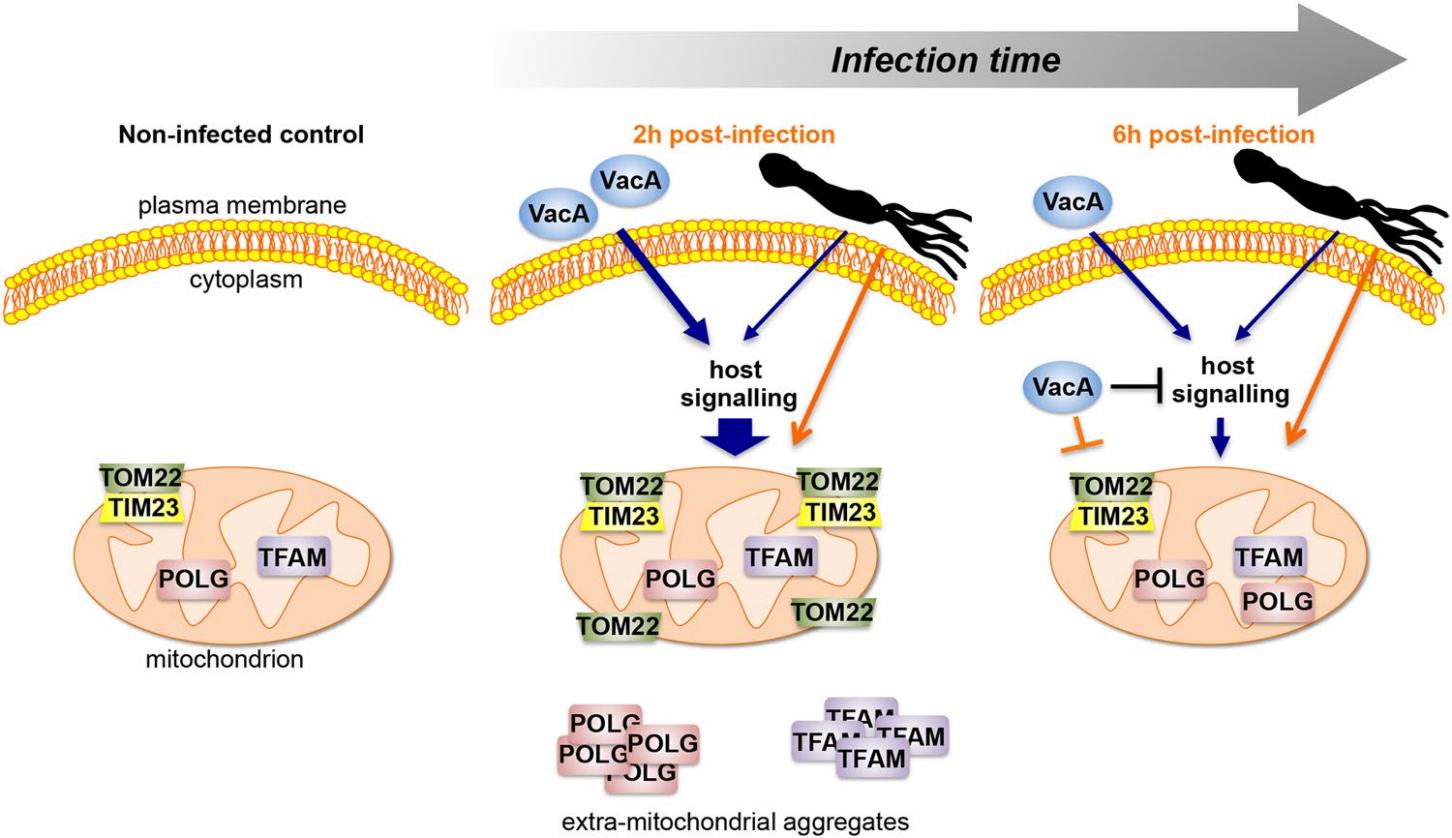
Schematic summary of VacA-mediated and VacA-independent mitochondrial alterations upon *H*. *pylori* infection. Left panel: non-infected cells with regular levels and localization of translocases subunits TOM22 and TIM23, DNA polymerase POLG, and transcription and DNA maintenance factor TFAM. Middle panel: during the first 2 hours of infection, extracellular VacA interacts with the epithelial cell surface activating host signaling pathways (we cannot exclude that undetectable levels of the VacA are present intracellularly and may also affect the levels of mitochondrial factors). This signaling results in enriched (large blue arrow) mitochondrial translocase subunits, in particular TOM22, dramatically induced POLG and TFAM, and doubled mtDNA content (not shown). At this stage, POLG and TFAM are largely extramitochondrial. These events are transitory, and are reduced or return to control levels (6 h pi, right panel) in the presence of intracellular VacA, which may inhibit (a blocked black line) and/or counteract (a blocked orange line) the signal(s) leading to mitochondrial alterations. TOM22, TIM23, POLG, and TFAM increase also in the absence of VacA, although at a lower extent, suggesting the involvement of unknown *H*. *pylori* activities (indicated with a schematic bacterium), not yet identified. These *H. pylori* activities may act through the same host signaling as VacA and/or through other interactions (orange arrow). VacA-independent alterations induce mitochondrial biogenesis with no increase of mtDNA content (not shown), and POLG and TFAM levels that remain higher than controls, suggesting a delayed effect than in the presence of VacA. At 48 h (not shown) and independently of the presence of VacA, TOM22, TFAM, POLG return to control levels, eventhough mitochondrial mass and mitochondria fragmentation increase and mtDNA is depleted.

*In vitro*, *H*. *pylori* displayed the most drastic effects on mitochondria after 2 h, when VacA was not detected intracellularly. Mitochondria were dramatically enriched in TOM22 and to a minor extent TIM23 translocase subunits, as illustrated in Fig. 7, without apparent changes of the mitochondrial mass. Imbalanced translocases likely affect the import of the key mitochondrial components POLG and TFAM, which were in large part localized externally to mitochondria, a condition that may in turn stimulate the production of these proteins. Alternatively, *H*. *pylori* may primarily affect POLG subcellular localization, thereby activating the mitochondrial import machinery to promote POLG internalization into mitochondria.

Our findings suggest that extracellular VacA or very few amounts of this protein inside the cell (which are undetectable by immunofluorescence) are sufficient to induce early mitochondrial alterations in infected cells. Thus, these effects may not require the internalization of VacA into mitochondria, nor its vacuolating activity. They could be due to the interaction of VacA with the gastric epithelial cells surface leading to activation of host signaling pathways, resulting in altered import of nuclear-encoded proteins into mitochondria (see scheme in Fig. 7). Accordingly, VacA binds various receptors at the epithelial and immune cells surface, triggering the pro-inflammatory response^38^ and activating signaling cascades, such as the β catenin pathway, which result in malignant cell transformation^38,39^. Of interest, VacA-induced activation of the pro-inflammatory p38/ATF-2 signaling cascade is independent of the cytotoxin endosomal and mitochondrial pathways^40^.

VacA itself requires TOM complex components (TOM20 and TOM40) to be imported into the mitochondrial inner membrane and form anion-channels^16^. Once VacA was detected intracellularly, TOM22 and TIM23 returned to control levels, as POLG and TFAM did, further underscoring the link between mitochondrial translocases and the levels of mitochondrial proteins.

The involvement of VacA in early up-regulation of TOM22, POLG and TFAM was also demonstrated upon exposure of cells to purified VacA(wt) protein. The N-terminal hydrophobic domain of VacA and its vacuolating activity were, however, not required for this regulation. Remarkably, the presence of VacA-(∆6-27), although not detected intracellularly, was sufficient to increase TOM22, POLG, and TFAM levels, as VacA did in *H*. *pylori*-infected cell, the protein not being detected intracellularly also in this case.

A further novel finding concerns VacA-independent mitochondrial deregulation, observed in *H*. *pylori* 26695∆*vacA*-infected cells, where TOM22, TIM23, POLG and TFAM were induced shortly upon infection, although at a lower extent and delayed compared to 26695 infection. These results support the involvement of yet unidentified *H*. *pylori* activities (a schematized bacterium in Fig. 7). Intracellular VacA may be able to antagonize these *H*. *pylori* activities, thereby explaining the higher amount of POLG at 6 h pi in *H*. *pylori* 26695∆*vacA- versus* 26695-infected cells.

Remarkably, up-regulation of POLG and TFAM in 12 months *H*. *pylori* SS1-infected INS-GAS mice occurred in parallel with exacerbation of the severity of gastric lesions, namely dysplasia and GIN development. Long-term *H*. *pylori* infection also induced mitochondrial biogenesis and deregulated mitochondrial translocases. The SS1 strain carries a VacAs2m2 cytotoxin with little if any cytotoxic activity. Up-regulation of POLG and TFAM were also observed in INS-GAS mice infected for 6 and 12 months with the *H*. *pylori* strain B128, which carries the impaired VacAs1m2 cytotoxin^41^(data not shown). Therefore, the late POLG and TFAM up-regulation *in vivo* could likely be VacA-independent, as observed at late infection time-points *in vitro*. Under these conditions, which were associated with the induction of catalase, suggesting oxidative stress generated by gastric inflammation, the mtDNA was largely depleted in the gastric mucosa of infected mice. These findings are in agreement with previous reports *in vitro* ^21^, with our data on late *H*. *pylori* infection in AGS cells (this study), and with studies in gastric biopsies from gastric cancer patients compared to gastritis patients^42^. The depletion of mtDNA could result from mutations, especially in the mtDNA regulatory region (D-loop)^43^ as we observed *in vitro* ^20^ and in *H*. *pylori*-infected mice^9^, or from dysfunction of the mitochondrial replisome (this study).

Importantly, our data were consistent through different infection paradigms (*in vitro* and *in vivo*), and with a variety of *H*. *pylori* strains, supporting the robustness of these observations. Acute interaction of gastric cells with bacteria or with purified VacA in *in vitro* culture is not strictly comparable with chronic interaction in the stomach not only in terms of paradigms but also of time-scales. Indeed, the effects that we report following infection in culture are temporary (they occur mainly early upon infection), and disappear with time, essentially allowing cells to proliferate. We consider these temporary effects as physiological to the infection, at least under our conditions. We reason that, however, upon repetitive contact with the bacteria, like during chronic infection *in vivo*, the infected tissues undergo repetitive stimuli and may cumulate alterations or improper responses, as the mitochondrial alterations described here. These responses may amplify with time and finally result in relevant effects on the long-term.

In conclusion, our findings reveal a novel and early inducer effect of *H*. *pylori* infection on mitochondrial translocases and the mtDNA replication/transcription machinery components POLG and TFAM. This effect is transient and its extinction is associated with cell survival to infection, although no causal effect between mitochondrial alterations and cell survival has been demonstrated. VacA is involved early in this process, possibly by interacting with the cells surface through activation of a host signaling cascade. Moreover, we show that VacA does not account for all consequences of *H*. *pylori* infection at mitochondria, pointing to the involvement of other bacterial activities, yet to be determined. These effects of *H*. *pylori* infection are also relevant *in vivo*, suggesting that mitochondrial alterations impact *H*. *pylori*-induced gastric inflammation and pathogenicity. Our study opens new investigative pathways to decipher the role of mitochondrial deregulation during host pathogens-interaction, and its impact in pathogenesis.

## Methods

### Ethics Statement

Mice experiments were carried out in strict accordance with the recommendations in the Specific Guide for the Care and Use of Laboratory Animals of the Institut Pasteur, according to the European Directives (2010/63/UE) and the corresponding French law on animal experimentation (arrêtés de 1988). The protocol has been approved by the Committee of Central Animal Facility Board of the Institut Pasteur. To follow the new European directives, the project was approved by the CETEA, Comité d’Ethique en Expérimentation Animale of the Institut Pasteur (Ref 2013-0051) and submitted for final approval to the Ministère de l’Enseignement Supérieur et de la Recherche (Ref 0031702).

### Cell culture, bacterial strains, and infection

Human gastric epithelial cells (AGS gastric adenocarcinoma cells: CRL1739-ATCC) were maintained in RPMI medium (Invitrogen), with 10% decomplemented fetal bovine serum and 1% penicillin-streptomycin (Invitrogen). *In vitro*, cells were infected with the *H*. *pylori* strain 26695 (ATCC 700392) which carries a fully active VacAs1m1 cytotoxin^41^, and the isogenic mutant deleted for *vacA* (this study, see Supplemental Experimental Procedure) for 2 h, 6 h, 24 h and 48 h, at a multiplicity of infection (MOI) of 100.  *H*. *pylori* strains were grown on blood agar base 2 (Oxoid) plates supplemented with 10% defibrinated horse blood incubated at 37 °C under microaerophilic conditions using an Anoxomat (MART Microbiology).

Total DNA and RNA from AGS cells were prepared as previously reported^20^ and used for the quantification of mtDNA by quantitative polymerase chain reaction (qPCR) and the level of gene expression by real-time reverse transcription polymerase chain reaction (RT-qPCR), respectively. Detailed methods for quantification of mtDNA by qPCR, and genes expression by RT-qPCR, Western blotting (WB), analysis of cell proliferation and apoptosis, immunofluorescence staining and Mitotracker® Deep-Red (LifeTechnologies) mitochondria labelling, three-dimensional (3D) imaging acquisition and quantification and modified fluorescence *in situ* hybridization (mTRIP)^30^ are described in the Supporting information.

### Incubation of AGS cells with VacA wild-type and VacA-(∆6-27) protein

Purified wild-type (wt) VacA protein from *H*. *pylori* strain 60190, VacA(wt) and VacA-(∆6-27) deleted for the N-terminal hydrophobic segment^34^ were prepared as previously described^44^. The *H*. *pylori* strain 60190, which carries the same VacAs1m1 cytotoxin as 26695, has the advantage to produce high amounts of VacA. Cells were incubated with 1µg of acid-activated VacA or VacA-(∆6-27) as reported^35^, for 2 h, 6 h, 24 h (4.10^5^ cells/well), and 48 h (2 × 10^5^ cells/well). Control cells were incubated with VacA activation buffer.

### Infection of mice

The *H*. *pylori* strain 26695 used *in vitro* is unable to colonise the gastric mucosa of mice. Six-weeks old INS-GAS/FVB male mice^23^ (n = 14) were orally inoculated with the *H*. *pylori* strain SS1^24^ (10^8^ colony-forming units (cfu)/100µl) at days 1 and 7; non-infected mice (n = 14) received peptone broth. After 6 and 12 months mice were sacrificed (n = 7/group). Gastric tissues were collected for histopathological and immunofluorescence analysis, DNA and RNA extracted as previously described^10^ (see Supporting information).

### Statistical analysis

Thirty cells (or fifty cells, when indicated) analysed in 3D/condition from three independent experiments were examined in immunofluorescence and MitoTracker experiments with cell cultures; data are presented as mean ± SD (standard deviation) of the three experiments. qPCR and RT-qPCR in cell cultures were performed on three independent cultures (with each meaurement done in duplicate), and results are expressed as mean ± SD of the three experiments. qPCR and RT-qPCR were performed on combined 7 mice per group, and each group was tested in three independent experiments (with each measurement done in triplicate), and results are expressed as mean ± SD of three experiments. Comparison of histology scores grading for inflammatory lesions and preneoplasia in the gastric mucosa between infected and non-infected mice were performed including 7 mice per group. For mice immunofluorescence analysis 750 mucous cells, 100 parietal cells and 750 chief and other cells were examined *per* condition, and the imunfluorescence/area was assessed to normalize for the variable size of different cell types. Differences between groups in immunofluorescence experiments were assessed by the unpaired t-test with the Welch’s correction (Welch’s test) for each condition as a source of three independent experiments per condition (significant differences were also confirmed by the Mann-Whitney test assuming equal SDs, but only the Welch’s test results are shown). VacA levels (in cells infected with *Hp*26695 or treated with isolated VacA) were assessed at the different time points with the Kruskal-Wallis test (Dunn’s multiple comparison tests) and ordinary one-way ANOVA, with compatible results. Differences in RT-qPCR and qPCR were assessed with one-way ANOVA. Differences in mitochondrial fragmentation were assessed with the two-tailed Fisher’s exact test. The two-tailed Welch’s t test was used when indicated. Statistical analyses were performed with GraphPad Prism 7.0.

## Electronic supplementary material

Supplementary Information
