# Supplementary material for: Helicobacter pylori targets mitochondrial import and components of mitochondrial DNA replication machinery through an alternative VacA-dependent and a VacA-independent mechanisms
Source: Sci Rep. 2017 Nov 21;7:15901. doi: 10.1038/s41598-017-15567-3 (PMC5698309; doi:10.1038/s41598-017-15567-3)

***Helicobacter pylori* targets mitochondrial import and components of mitochondrial DNA replication machinery through an alternative VacA-dependent and a VacA-independent mechanisms**

Laurent Chatre, Julien Fernandes, Valérie Michel, Laurence Fiette, Patrick Avé, Giuseppe Arena, Utkarsh Jain, Rainer Haas, Timothy C Wang, Miria Ricchetti and Eliette Touati

**Supplementary Information**

**Material and methods**

***Construction of H. pylori 26695 ∆VacA mutant***

Chromosomal inactivation of *hp0887* (encoding VacA) in the *H. pylori* strain 26695 was performed by allelic exchange using a recombinant plasmid derived from PCR8/GW/TOPO (Invitrogen) in which was subcloned a PCR-amplified fragment carrying ≈ 450bp of the *hp0887* 5’- and 3’- flanking regions*.* This fragment, which overlaps with *hp0886* (5’) and *hp0888* (3’), and carries a kanamycin resistance cassette, was amplified using the following primers: HP26F0886, 5’CTAAAAACAAGGCTTTAAAGGGCG3’; HP26K2R0886, 5’CATTTATTCCTCCTAGTTAGTGGTGCGACTTTAGACTAGAAAATTTCTGTTGC3’; HP26K2F0888, 5’TACCTGGAGGGAATAATGATACCGCTCTTAAACCCATGCTCAAAGCATGG3’ and HP26R0888, 5’GCTATGCCTAAAGCTAAGCAC3’. The mutant strain ∆*vacA* was obtained by natural transformation of *Hp*26695 as previously described^1^, using 1µg of the PCR fragment and selecting for kanamycin resistance (20µg/ml). The construction was confirmed by PCR analysis and nucleotide sequencing.

***Immunostaining of cells and mice gastric tissue sections***

Cells were cultured on slides, fixed with 2% PFA and permeabilized with 0.5% Triton X-100. The slides were incubated in blocking buffer (BSA 5% in phosphate buffer saline (PBS) for 1h prior incubation with the primary antibody (anti-TOM22 coupled Atto488 (Sigma-Aldrich), anti-POLG^2^, anti-TFAM^2^ and anti-PARP-1 (H-250, Santa Cruz Biotechnologies), anti-cleaved Caspase 3 (D-175, Cell Signaling, ref 9661)^3,4^, anti-cleaved Caspase-6 (Cell Signaling, ref. 9761), LC3B (Sigma-Aldrich), NF-kB p105/p50 and NF-kB p65 from Abcam. A secondary anti-mouse or anti-rabbit antibody Alexa® Fluor 555 or Alexa® Fluor 488 conjugated (Invitrogen) was applied^2^. The polyclonal rabbit anti-VacA antibody was obtained from R. Haas laboratory^5,6^. The nuclear DNA was stained with 10 μg/ml Hoechst 33342. MitoTracker Deep Red^3,4^ at a concentration of 200 nM was incubated with cells for 1h before fixation.

For mice gastric tissue sections, paraffin embedded sections were dewaxed with 2% liquid dish washing soap at 70°C for 10 min. Sections were incubated in blocking buffer (BSA 5% in PBS) for 1h prior overnight incubation with the primary antibody. A secondary antibody was applied for 1h with Hoechst 33342. MitoTracker Deep Red at a concentration of 200 mM was incubated with dewaxed sections for 1h. Mitotracker Deep red labelling was followed by TIM23 (Sigma_Aldrich, ref: HPA031408; validation: <http://www.proteinatlas.org/ENSG00000265354-TIMM23/antibody>) immunostaining (see procedure above). Imaging analysis was carried out using spinning-disk Perkin Elmer Ultraview RS confocal microscope. Labelling and image acquisitions of samples belonging to the same series (including different time points) were performed in parallel.

***Labeling and denaturation of mitochondrial DNA probes for mTRIP***

The mtDNA probes for mTRIP^2^ (mitochondrial Transcription and Replication Imaging Protocol), a fluorescence *in situ* hybridization analysis (see below), were labeled by nick translation of PCR products, incorporating either Atto488-dUTP or Atto550-dUTP (Jena Bioscience), using the commercial kit Atto488/Atto550 Nick Translation Labeling kit (Jena Bioscience), as previously described^2^. Forty ng of labeled probes were mixed with salmon sperm DNA (Sigma) and hybridization buffer (50% formamide, 10% dextran sulfate, in 2x saline-sodium citrate (SSC) buffer at pH 7.0). The hybridization mix was denaturated at 80°C for 10 min. Coordinates of primers are indicated according to NC_012920 GenBank, and primer sequences are indicated in ref^2^. Mitochondrial coordinates of mREP correspond to the region 446-544, and mTRANS to the coordinates of three distinct probes (1905-2866, which covers part of *16S rDNA*; 7400-8518, which extends from *COI* end to *ATP8;* and 13416-14836 from *ND5* end until the beginning of *CYTB*)^2,4^.

***Probe hybridization and mTRIP protocol***

Cells were cultured on slides, fixed and permeabilized as described above (see immunostaining), then incubated in 50% formamide (pH=7.0)/2x SSC (saline-sodium citrate buffer) and denaturated in 70% formamide/2x SSC. Hybridization with the mREP and mTRANS probes was performed for 16h at 37°C. After washing the slides in 2x SSC, 1x SSC, then 0.1x SSC, DNA was stained with 10 μg/ml Hoechst 33342 and imaging analysis was carried out as described in the next section.

***Confocal acquisition, 3D-reconstruction and quantification***

Confocal acquisition was performed using a spinning-disk Perkin-Elmer Ultraview RS Nipkow Disk, an inverted laser-scanning confocal microscope Zeiss Axiovert 200M with an Apochromat 63x/1.4 oil objective and a Hamamatsu ORCA II ER camera (Imagopole, CiTech, Institut Pasteur), as described^2,7^. Briefly, images were acquired using non-saturating settings, and the same imaging parameters were used for all samples. Optical slices were taken every 200-nm interval along the z-axis covering the whole depth of the cell, at resolution of 1024/1024 pixels. All acquisitions were post-treated using the same Intensity Range (Min/Max), Maximum Intensity Projection, and background correction using the Imaris software (Bitplane), which automatically performs pixel averaging. Three-dimensional (3D)-reconstruction was achieved using the IMARIS software. Original 2D images generated from 3D volume rendering were used for fluorescence quantification using a single-imaging frame collection and ImageJ 1.34-s software (post-acquisition analysis). Graphic settings were applied to 2D images for the purpose of visualization (panels in figures). Co-localization studies were performed with ImageJ JACoP plug-in. For each condition, thirty 3D-reconstructed cells were analysed from three independent experiments. p ≤ 0.05*; p ≤ 0.01**; p ≤ 0.001***; p ≤ 0.0001****; *versus* control non-infected, based on the unpaired Welch’s test.

***Analysis of cell proliferation and apoptosis***

To analyse cell proliferation under the conditions of *H. pylori* infection used in this study, cells were cultured in 96-well plates at 40% and 80% confluence for infection time point at 24h and 48h, respectively. *H. pylori* infection was performed for 24h and 48h at MOI 100. Cell proliferation was assessed by a colorimetric immunoassay based on the measurement of BrdU (5’-Bromo-2’-deoxyuridine) incorporation during DNA synthesis, using the Cell Proliferation ELISA, BrdU assay (Roche Applied Science). The reaction product was quantified by measuring the absorbance with a scanning multi-well spectrophotometer (ELISA reader) at 450nm. Apoptosis was analysed by flow cytometry using Annexin V staining. The cell cycle distribution was analysed with propidium iodide (PI) staining and analysis by flow cytometry. FACS (fluorescence-activated cell sorting). For cell cycle and apoptosis experiments, samples were analyzed on a Becton-Dickinson FACS Calibur (BD Biosciences) flow cytometer. All FACS data were analysed with FlowJo v9 software (Tree Star, USA) for cell cycle distribution and FITC staining of cells.

***Quantification of mitochondrial DNA by real-time polymerase chain reaction (qPCR)***

The quantification of mtDNA was done as described^8^. Realtime PCR amplification was performed on 200 pg of total DNA using the StepOne^TM^ Plus RealTime PCR system (Applied Biosystems) and Power Sybr Green PCR Master mix (Life Technologies), following the manufacturer’s instruction. In human gastric epithelial cells (AGS), a 211 bp fragment of the mtDNA *12S rRNA* gene was amplified (nucleotides, 1095-1305, human mitochondrial DNA accession [http://www.mitomap.org/NC012920.1]). The nuclear encoded *18S rRNA* gene was used as endogenous reference. In mice, DNA was isolated from the gastric mucosa as previously described^9^. A fragment of the mitochondrial *COI* gene was amplified, and the nuclear encoded *NDUFV1* gene was used as endogenous reference^10^. The level of mtDNA was calculated using the ΔC_T_ of average C_T_ of mtDNA and nDNA (ΔC_T_ = C_T_ nDNA - C_T_ mtDNA) as 2^ΔCT 11^. Primers used for amplification are described in Table S1.

***Quantification of gene expression by quantitative reverse transcriptase coupled to polymerase chain reaction (RT-qPCR)***

Total RNA was isolated either from AGS cells using a RNeasy Mini kit (Qiagen) or from mice stomach as previously described^9^, and purified on RNeasy mini columns (Qiagen). RNA was treated with DNaseI (Qiagen), reverse-transcribed using Superscript® III Reverse transcriptase (Invitrogen) and amplified with Power Sybr Green PCR Master Mix (Applied Biosystems) using the StepOne^TM^ Plus RealTime PCR system (Applied Biosystems). Target genes were normalized according to the TATA-box-binding protein gene (TBP). Amplification PCR consisted in 40 cycles of 15 seconds at 95°C and 1 min 60°C. Data were analyzed by StepOne Plus RT-PCR software v2.1. Primers used for amplification are listed in Table S1.

***Detection of proteins level by Western blot***

Total protein extracts were prepared from AGS cells co-cultured with *H. pylori* strains or treated with purified wild-type VacA or VacA-(∆6-27) proteins, resuspended in lysis buffer [50 mM Tris-HCl pH 7.5, 150 mM NaCl, 1% Triton X-100, 0.1% SDS, 1mM EDTA, and protease inhibitor cocktail (Roche)]. Lysed cells were not centrifuged and the whole extract was collected. The protein content was determined with the Bradford reagent (Sigma-Aldrich), and 20 µg of protein were loaded for SDS-PAGE. After blotting, Hybond ECL nitrocellulose filters were probed with primary antibodies, then with IRDye secondary antibodies. Detection was performed using Odyssey Infrared Imaging system scanner and Odyssey application software v3.0 (LI-COR Biosciences, Lincoln, NE). For experiments in Fig. S3D, anti-rabbit HRP-conjugated secondary antibodies (Cell Signaling) were used and detection was performed by using either Pierce™ ECL Western Blotting Substrate or SuperSignal™ West Femto Maximum Sensitivity Substrate (Thermo Fisher Scientific). For experiments with cells in culture, the proteins charge loading on gels was determined by Sypro Ruby (Invitrogen) staining^12,13^ or GAPDH immunostaining when the levels of this marker were stable among samples. For experiments on gastric extracts, GAPDH was used as internal control of protein loading since, differently from experiments with cells in culture, the levels of this markers were stable amongst tested conditions.

***Production and genotyping of INS-GAS mice***

Five-6 weeks old specific pathogen free INS-GAS/FVB male mice^14,15^ (n=28) obtained from an in-house breeding colony initiated from 4 couples from TC Wang laboratory, Columbia University College, New-York, NY, USA, were used. The presence of the INS-GAS trans-gene was confirmed by genotyping on DNA extracted from mouse tail and amplified by PCR using the following primers: Forward: 5’ TGATCTTTGCACTGGCTCTG3’ and Reverse: 5’TCCATCCATCCATAGGCTTC3’.

***Histopathology analysis of gastric lesions in mice***

Stomach samples from non-infected and infected mice were fixed in RCL2® (Alphelys, France) and embedded in low-melting-point paraffin wax (Poly Ethylene Glycol Distearate; Sigma, USA). Four µm-thick sections were stained by hematoxylin and eosin treatment (H&E) and Periodic acid Schiff-Alcian Blue and examined blindly for histopathologic lesions as already reported^9^. Histologic alterations (i.e. inflammation, ulceration, foveolar hyperplasia, intestinal metaplasia, parietal cell loss, dysplastic changes of the gastric mucosa and herniation), which were semi-quantitatively evaluated based on a scoring system with five severity grades (1: minimal, 2: mild, 3: moderate, 4: marked and 5: severe) were characterized as previously described^16,17^.

***Determination of mtDNA mutations in the gastric mucosa of mice***

DNA was isolated from the gastric mucosa of infected and non-infected mice as already described^9^. An equal proportion of DNA from 7 mice per group was pooled and amplified by PCR using primers mitDm5: 5’CATTAAACTATTTTCCCCAAGCA3’ and mitDm3: 5’GAGTTTTGGTTCACGGAAACATGA3’ at nucleotides 41 and 843 in the mtDNA D-loop sequence, respectively [http://www.genome.jp/NC_005089]. The amplified fragments from DNA isolated from infected and non-infected mice, were cloned in *Escherichia coli* using the pDrive vector (In vitrogen) and then sequenced (GATC Biotech). Sequencing data were analysed using DNAstar software. The mutant frequency was determined by the ratio of the number of *E. coli* clones with a mutation in the mtDNA sequence on the total number of sequenced clones.

**References**

1 Bury-Mone, S. *et al.* Presence of active aliphatic amidases in Helicobacter species able to colonize the stomach. *Infect Immun* **71**, 5613-5622 (2003).

2 Chatre, L. & Ricchetti, M. Large heterogeneity of mitochondrial DNA transcription and initiation of replication exposed by single-cell imaging. *J Cell Sci* **126**, 914-926, (2013).

3 Rocheteau, P. *et al.* Sepsis induces long-term metabolic and mitochondrial muscle stem cell dysfunction amenable by mesenchymal stem cell therapy. *Nat Commun* **6**, 10145, (2015).

4 Chatre, L. & Ricchetti, M. mTRIP: an imaging tool to investigate mitochondrial DNA dynamics in physiology and disease at the single-cell resolution. *Meth Mol Biol (Clifton, N.J.)* **1264**, 133-147 (2015).

5 Vinion-Dubiel, A. D. *et al.* A dominant negative mutant of Helicobacter pylori vacuolating toxin (VacA) inhibits VacA-induced cell vacuolation. *J Biol Chem* **274**, 37736-37742 (1999).

6 Sewald, X., Jimenez-Soto, L. & Haas, R. PKC-dependent endocytosis of the Helicobacter pylori vacuolating cytotoxin in primary T lymphocytes. *Cell Microbiol* **13**, 482-496, (2011).

7 Chatre, L., Montagne, B. & Ricchetti, M. A Single-Cell Resolution Imaging Protocol of Mitochondrial DNA Dynamics in Physiopathology, mTRIP, Which Also Evaluates Sublethal Cytotoxicity. *Meth Mol Biol (Clifton, N.J.)* **1351**, 49-65 (2016).

8 Parone, P. A. *et al.* Preventing mitchondrial fission impairs mitochondrial function and leads to loss of mitochondrial DNA. *PLoS One* **3**, s (2008).

9 Touati, E. *et al.* Chronic Helicobacter pylori infections induce gastric mutations in mice. *Gastroenterology* **124**, 1408-1419 (2003).

10 Guo, W., Jiang, L., Bhasin, S., Khan, S. M. & Swerdlow, R. H. DNA extraction procedures meangfully influence qPCR-based mtDNA copy number determination. *Mitochondrion* **9**, 261-265 (2009).

11 Xia, P. *et al.* Decreased mitochondrial DNA content in blood samples of patients with stage I breast cancer. *BMC Cancer* **21**, 454 (2009).

12 VanMeter, A. J. *et al.* Laser capture microdissection and protein microarray analysis of human non-small cell lung cancer: differential epidermal growth factor receptor (EGPR) phosphorylation events associated with mutated EGFR compared with wild type. *Mol Cell Proteomics* **7**, 1902-1924 (2008).

13 Steinberg, T. H. *et al.* Rapid and simple single nanogram detection of glycoproteins in polyacrylamide gels and on electroblots. *Proteomics* **1**, 841-855 (2001).

14 Wang, T. *et al.* Synergistic interaction between hypergastrinemia and Helicobacter infection in a mouse model of gastric cancer. *Gastroenterology* **118**, 36-47 (2000).

15 Fox, J. *et al.* Helicobacter pylori associated gastric cancer in INS-GAS mice is gender specific. *Gut* **51**, A47 (2002).

16 Rogers, A. B. *et al.* Helicobacter pylori but not high salt induces gastric intraepithelial neoplasia in B6129 mice. *Cancer Res* **65**, 10709-10715, (2005).

17 Eaton, K., Radin, M. & Krakowka, S. An animal model of gastric ulcer due to bacterial gastritis in mice. *Vet Pathol* **32**, 489-497 (1995).

18 Arena, G. & Valente, E. M. PINK1 in the limelight: multiple functions of an eclectic protein in human health and disease. *Journal Pathol* **241**, 251-263, (2017).

19 Mercy, L. *et al.* Mitochondrial biogenesis in mtDNA-depleted cells involves a Ca2+-dependent pathway and a reduced mitochondrial protein import. *The FEBS journal* **272**, 5031-5055 (2005).

20 Squires, J. E., Stoytchev, I., Forry, E. P. & Berry, M. J. SBP2 binding affinity is a major determinant in differential selenoprotein mRNA translation and sensitivity to nonsense-mediated decay. *Mol Cell Biol* **27**, 7848-7855 (2007).

21 Suissa, S. *et al.* Ancient mtDNA genetic variants modulate mtDNA transcription and replication. *PLoS Genet* **5**, e1000474 (2009).

22 Cooper, M. P. *et al.* Defects in energy homeostasis in Leigh syndrome French Canadian variant through PGC-1alpha/LRP130 complex. *Genes Dev* **20**, 2996-3009, (2006).

23 Bhusari, S., Hearne, L.B., Spiers, D.E., Lamberson, W.R., Antoniou, E., . Transcriptional profiling of mouse liver in response to chronic heat stress. *J Therm Biol* **33**, 157-167 (2008).

24 Choi, J. *et al.* Mst1-FoxO signaling protects Naive T lymphocytes from cellular oxidative stress in mice. *PLoS One* **4**, e8011 (2009).

25 Bonnard, C. *et al.* Mitochondrial dysfunction results from oxidative stress in the skeletal muscle of diet-induced insulin-resistant mice. *J Clin Invest* **118**, 789-800, (2008).

**Supplementary figure legends**

**Figure S1. Long-term *H. pylori* infection induces chronic inflammation and oxidative stress associated with the development of gastric intraepithelial neoplasia (GIN) in INS-GAS mice.** Mice were chronically infected by oral gavage with the *H. pylori* strain SS1 for 6 and 12 months, and gastric lesions were compared to non-infected mice. **A)** Representative histological changes in the stomach of *H. pylori* infected mice (b, d, f, h) and non-infected mice (a, c, e, g) after 6 months (a, b, e, f) and 12 months (c, d, g, h) pi on hematoxylin and eosin (H&E) (a to d) and Periodic acid-Schiff and Alcian (PAS-Alcian) Blue (e to h) stained paraffin sections. Original magnification 2x, bar: 500 μm. Non-infected mice after 6 months show gastritis, epithelial defects with glands dilatation, glandular hyperplasia, and parietal gland atrophy with intestinal metaplasia as indicated by the presence of intestinal type acidic mucin (blue) or mixed mucins (azure) instead of gastric-type neutral mucins (red) on the PAS-Alcian Blue staining, and dysplasia (a, e). Infected mice, at 6 and 12 months pi show more severe inflammatory (*p=0.048 and p=0.002*), atrophy (*p=0.005 and p=0.0005*), hyperplasia (*p=0.048 and p=0.002*), intestinal metaplasia (*p=0.05*; 6 months*)* and dysplasia (*p=0.012 and p=0.003*), associated with a greater thickness of the gastric mucosa compared to controls (b, f). Uninfected mice show more severe gastric lesions after 12 months than 6 months (c, g). After 12 months, infected-mice display low-grade GIN, as defined by dysplasia and herniation into the sub-mucosa (d, h). **B)** Anti-*H. pylori* antibodies in the sera of mice detected by Enzyme-linked Immunosorbant Assay (ELISA). The results are presented as A405-492 readings for diluted samples (1:100). Horizontal bars represent median values. **C)** Semi-quantitative evaluation of histologic lesions induced by *H. pylori* infection in the gastric mucosa of mice. The microscopic changes were characterized on gastric tissue sections stained by (H&E) and PAS-Alcian blue staining. Scores for the severity of gastric inflammation, presence of hyperplasia, intestinal metaplasia and dysplasia, which are considered preneoplastic lesions, and atrophy of parietal cells are similar after 6 and 12 months of infection and higher than the non-infected group at the same time-point. Infected *vs* non-infected mice, p<0.05*; p<0.01**; p<0.001***. **D)** Expression of genes coding for *Sod1*, *Sod2,* and *Catalase* quantified by RT-qPCR from RNA isolated from the gastric mucosa of infected and non-infected mice at each time-point. Values represent the mean ± SD of three independent measurements for each group of mice. Infected mice compared to non-infected; p<0.05*, p<0.01**.

**Figure S2. Apoptosis, autophagy, and NF-κB factors in mice gastric mucosa upon 12-month infection.** Western blot analyses of gastric sample extracts of three independent mice per condition (6-month and 12-month old mice, untreated or infected with the *Hp*SS1 strain). **A)** Infection results in no change or increase of one of the two large fragments (p17/p19) of cleaved Caspase-3 in two out of three mice per condition. The levels of the housekeeping protein GAPDH, as well as the total protein load stained with Ponceau S red in the selected area are shown below. Samples were derived from the same experiment and the gel and blots were processed in parallel. The blot was then sliced in horizontal sections to allow multiple immunolabelings with the same membrane. Sections were alternatively labeled with anti-cleaved Caspase-3 and anti-GAPDH. The corresponding uncropped gel/blots are shown in Supplementary Figure S7E. **B)** Upper panel, increased levels of the p50 factor (canonical NF-κB) but not of the p105 precursor, nor of the p65 (relA) activator 12 months after infection compared to non-infected mice. Lower panel, increased levels of one or both LC3B bands after 12 months infection compared to non-infected mice. Samples were derived from the same experiment and the gels and blots were processed in parallel. Each blot was then sliced in horizontal sections to allow multiple immunolabelings with the same membrane. The same gel was used for the p105/p50, p65, and LC3B analyses; the levels of the housekeeping protein GAPDH are shown below. The corresponding uncropped gel/blots are shown in Supplementary Figure S7F (the original, uncropped p105/p50 blot is shown here).

**Figure S3. Cell proliferation and apoptosis upon *H. pylori* infection of AGS cells. A)** Cell proliferation was measured by quantification of BrdU incorporation in the DNA of AGS cells infected with *Hp*26695 and *Hp*26695∆*vacA* for 24h (upper panel) and 48h (lower panel). The analysis was performed during 48h in cultures initiated with 80% and 40% confluent cells for the time-points 24h and 48h pi, respectively. Note that in the upper panel we extended analysis beyond the 24h time-point; both non-infected and infected cells proliferate in the next 24h (proliferation); p<0.001* 48h *vs* 24h. When not visible, error bars stand inside the size of the symbols**.** Apoptosis was detected by flow cytometry using Annexin V-FITC fluorochrome at 24h (upper panel) and 48h (lower panel). **C)** Analysis of the cell cycle with PI and FACS of non-infected and infected cells at 24h (upper panel) and 48h (lower panel). Histograms show the percentage of cells in each of the G0/G1, S, and G2/M phases of the cell cycle, after doublet discrimination. Mean of 3 experiments ± SD. **D)** Western blot of PARP-1, full-length and cleaved form (89 kDa, apoptosis), shows signal in all samples, including non-infected cells. The percent of cleaved PARP-1, which is digested by caspases (in particular Caspase-3) and other enzymes is shown in the histogram on the right. On the right the positive control C1+ (H1299 cells treated with 100µM Menadione for 1h). Note high basal levels of cleaved PARP-1 in AGS cells. The blot was sliced in horizontal sections to allow multiple immunolabelings of the same membrane (the housekeeping protein GAPDH, cleaved Caspase-3 and cleaved Caspase-6, which are shown below). The cleaved Caspase-6 labelling was performed on the same membrane as for cleaved Caspase-3, after stripping from the previous labelling. No detectable cleaved Caspase-3 (17 kDa-19 kDa) or cleaved Caspase-6 (prevalent band 18 kDa) signal was observed in AGS cells either non-infected or infected with the *Hp26695* and *Hp26695∆VacA* strains from 2h to 48h, differently from the positive control (C2^+^, H1299 human cells transduced with PINK-1-shRNA for 5 days, an established model of activation of apoptosis^18^). **E)** Increased levels of both LC3B bands in particular form I) after 24h infection with the *Hp26695* and, to a minor extent with the *Hp26695∆VacA* strain. At 48h the levels of LC3B are undetectable as in controls. The blot was sliced in horizontal sections to allow multiple immunolabelings with the same membrane (only LC3B labeling is shown here). The levels of the housekeeping protein GAPDH are shown below. **F)** Increased levels of the p50 factor (canonical NF-κB) at to some extent of the p105 precursor, at 24h pi with the *Hp26695* strain, but not with the *Hp26695∆VacA* strain. These proteins, in particular p105, migrate slower than predicted from their size. For WB in E and F, as well as in D, samples were derived from the same experiments and the gels and blots were processed in parallel. The corresponding uncropped blots are shown in Supplementary Figure S7G-I. Western blots in panel D were detected with chemioluminescence (HRP-conjugated secondary antibodies).

**Figure S4. *H. pylori* mildly induces initiation of mtDNA replication and mitochondrial transcripts early upon infection. A)** Human mitochondrial genome map. Gene names are indicated outside the circle. tRNA genes are indicated by the amino acid corresponding letter. Genes coding for ribosomal RNAs 16S and 12S are in dark-grey and the D-loop region in black. In the inner circle are indicated the probes used in the mTRIP assay^2,4,7^. In red is indicated the position of mREP probe and in green the position of the three individual probes corresponding to mTRANS. **B)** Confocal 3D analysis of mTRIP labeling of AGS cells infected with *Hp*26695 and *Hp*26695∆*vacA* for 2h, 6h, 24h and 48h. Fluorescence labeling with mREP (red), mTRANS (green) probes, and merge (right panel). Nuclei are counterstained with Hoechst (blue). **C)** Quantification of the fluorescence intensity of mREP and mTRANS, performed on 30 cells from 3 independent experiments *per* condition (n=50 non-infected cells), mean ± SD, Welch’s test, p<0.05*, p<0.01**; p<0.001***; p<0.0001*****.* Note that accessible initiation of replication (mREP probe) and mitochondrial transcripts (mTRANS probe) at best doubled or were essentially not affected in *Hp*26695 and *Hp*26695∆*vacA*-infected cells compared to controls, with the exception of mTRANS that progressively declined from 24h, largely in a VacA-dependent manner. mTRANS reduction was concomitantly with TFAM return to control levels (see Fig 4E). **D)** Quantification by RT-qPCR of mRNA levels of the mitochondrial genes *16rRNA* and *CYTB* located at the beginning and at the end of the H strand, respectively. Note that transcription of these genes was almost unaffected by the infection, except a VacA-independent increase at 6h pi, as also observed for TFAM (see Fig. 4E). **E)** Ratio of *16S* transcripts over mTRANS (left panel) and *CYTB* transcripts over mTRANS (right panel), expressed in arbitrary units. mTRANS includes processed and unprocessed mitochondrial transcripts in single cells that are accessible to the probe, whereas RT-qPCR reveals processed transcripts in a cell population. Under normal culture conditions, RT-qPCR and mTRANS profiles have been shown to be similar^2^. However, *H. pylori* infection appears to occasionally enrich in processed transcripts over global transcripts (mTRANS). Reported values are the mean ± SD of three independent experiments. Infected *versus* non-infected cells; p<0.05*; p<0.01**; p<0.001***.

**Figure S5. Reduced antioxidant defence upon *H. pylori* infection is mildly affected by purified VacA.** Expression of genes coding for antioxidant *Sod1*, *Sod2,* and *Catalase* quantified by RT-qPCR from RNA isolated from **A)** infected and non-infected cells and **B)** VacA-treated and non-treated cells. Untreated: AGS cells; Mock: AGS cells incubated with the VacA activation buffer as control. Values represent the mean ± SD of three independent measurements for each condition. Comparison to non-infected (A) or to non-treated cells (B); p<0.001***.

**Figure S6. Purified VacA protein mildly induces initiation of mtDNA replication and mitochondrial transcripts. A)** Confocal 3D analysis of mTRIP labeling of AGS cells incubated for 2h to 48h with 1µg/ml of acid-activated VacA(wt) and VacA-∆(6-27), as previously described^5,6^. Untreated: AGS cells; Mock: AGS cells incubated with the VacA activation buffer as control. Initiation of mtDNA replication and mitochondrial transcripts are visualized by the mREP (red, three left panels) and mTRANS (green, three middle panels) probes, respectively; merge is shown on three right panels. Nuclei are visualized by Hoechst staining (blue). **B)** Early upon treatment VacA(wt) but not VacA-(∆6-27) increases mREP and mTRANS signal. mTRANS staining also increases in Vac(wt) treated cells after 24h. Quantification of the fluorescence intensity of mREP and mTRANS labelling was performed on 30 cells (n=50 untreated cells) from three independent experiments *per* condition, mean ± SD, Welch’s test, p<0.05*; p<0.01**; p<0.001***; p<0.0001****. Note that mTRIP analyses of VacA-treated cells were compatible with the results obtained from infected cells after 2h (in the case of VacA(wt) treatment) and 48h (VacA(wt) and VacA-(∆6-27) treatment) (see above, Fig S4). **C)** Quantification by RT-qPCR of mRNA levels of the mitochondrial genes *16rRNA* and *CYTB* located at the beginning and at the end of the H strand, respectively; no relevant effect of either VacA protein was observed. Reported values represent the mean ± SD of three independent experiments. Treated *versus* non-treated cells; p<0.001***.

**Figure S7. Original uncropped gels and blots for WB.** **A)** For reference, the largest molecular weight (MW) markers and their size (left) and POLG immunostaining (right) from a reference gel. **B)** Uncropped immunoblots from the lower panel in Figure 2C, shown in the order of their position on the uncut membrane. The membrane blot was horizontally sliced into three portions to allow multiple immunolabelings with the same membrane: upper portion, anti-POLG; middle portion, anti-GAPDH; lower portion, anti-TFAM). The molecular weight markers (and the corresponding size in kDa) are on the left of the membrane. For each membrane, the part shown in Figure 2C is indicated with a rectangle. **C)** Uncropped gel and immunoblots from Figures 4C and 4F. The original gels (one gel for 2h and 6h, the other gel for 24h and 48h) stained with SYPRO are shown in the upper part with the corresponding molecular weight markers. Rectangles indicate the horizontal slices that have been used for immunoblots, which are shown below. The upper membrane was immunostained with POLG, the lower membrane with TFAM. Rectangles on immunoblots indicate the parts shown in Figure 4C (POLG) and 4F (TFAM). **D)** Uncropped gel and immunoblots from Figures 6C and 6F. The original gels (one gel for 2h and 6h, the other gel for 24h and 48h) stained with SYPRO are shown in the upper part with the corresponding molecular weight markers. Rectangles indicate the horizontal slices that have been used for immunoblots, which are shown below. The upper membrane was immunostained with POLG, the lower membrane with TFAM. Rectangles on immunoblots indicate the parts shown in Figure 6C (POLG) and 6F (TFAM). For C) and D) images of SYPRO-stained gels were taken with a UV Gel Doc^TM^ Camera BioRad, whereas images of immunoblots were taken with the Odyssey Imager LI-COR. Images are shown from the original scale of each acquisition. **E)** Uncropped blots of samples from Supplementary Figure S2A. Rectangles indicate the parts shown in the figure. **F)** Uncropped blots of samples from Supplementary Figure S2B. Rectangles indicate the parts shown in the figure. The entire p105/p50 immunoblot was already shown in the figure. **G)** Uncropped blots of Supplementary Figure S3D. Cleaved Caspase-6 labelling was performed on the same membrane used for cleaved Caspase-3, successively, after stripping. Rectangles indicate the parts shown in the figure. Other samples (indicated with “x” are irrelevant to the present study). **H)** Uncropped blots of Supplementary Figure S3E. Rectangles indicate the parts shown in the figure. Material from the same experiment was used and load in one gel then used for LC3B immunolabelling and on another gel used for GAPDH immunolabelling, which were run and processed in parallel. **I)** Uncropped blot of Supplementary Figure S3F. The rectangle indicates the part shown in the figure.

| **RT-qPCR primers** | | | |
| --- | --- | --- | --- |
| **GENE** | **FORWARD** | **REVERSE** | **Reference** |
| **Human TBP** | 5'CTCACAGGTCAAAGGTTTAC | 5'GCTGAGGTTGCAGGAATTGA | *^18^* |
| **SOD1** | 5'GGTGGGCCAAAGGATGAAGA | 5'GGCGATCCCAATTACACCAC | *^19^* |
| **SOD2** | 5'CCTCCCCGACCTGCCCTACG | 5'TCTCCCTTGGCCAACGCCTC | *^19^* |
| **Catalase** | 5'ACTGGGATCTCGTTGGAAAT | 5'CCCCCGATCACTGAACAAGA | *^19^* |
| **RNA 16S** | 5'GTATGAATGGCTCCACGAGG | 5'GGTCTTCTCGTCTTGCTGTG | *^20^* |
| **CYTB** | 5'CTCCCGTGAGGCCAAATATC | 5'GAATCGTGTGAGGGTGGGAC | *^20^* |
| **Mouse TBP** | 5'CTTCACCAATGACTCCTATG | 5'TGACTGCAGCAAATCGCTTG | *^21^* |
| **SOD1** | 5'GGACCTCATTTTAATCCTCAC | 5'TGCCCAGGTCTCCAACATG | *^22^* |
| **SOD2** | 5'CAGACCTGCCTTACGACTATG | 5'CTCGGTGGCGTTGAGATTG | *^23^* |
| **Catalase** | 5'AGAGAGCGGATTCCTGAGAGA | 5'ACCTTTCCCTTGGAGTATCTG | *^23^* |
| **POLG** | 5'GCGGCTGGTGGAAGAGCGTT | 5'GAGATGGCCATGTGCATGCT | *^24^* |
| **TFAM** | 5'GCTTCCAGGAGGCAAAGGAT | 5'CCAAGCCTCATTTACAAGC | *^25^* |
|  |  |  |  |
| **qPCR primers** | | | |
| **GENE** | **FORWARD** | **REVERSE** | **Reference** |
| **Human RNA18S** | 5'GAGAAACGGCTACCACATCC | 5'GCCTCGAAAGAGTCCTGTAT | *^20^* |
| **RNA12S** | 5'GCTCGCCAGAACACTACGAG | 5'CAGGGTTTGCTGAAGATGGC | *^7^* |
| **Mouse NDUFV1** | 5'CTTCCCCACTGGCCTCAAG | 5'CCAAAACCCAGTGATCCAGC | *^8^* |
| **CO1** | 5'TGCTAGCCGCAGGCATTAC | 5'GGTGCCCAAAGAATCAGAAC | *^8^* |

**Table S1: Primers used in this study**

| **TOM22** | | | |
| --- | --- | --- | --- |
|  | Non-infected | *Hp 26695* | *Hp 26695∆vacA* |
| **2h**  **6h**  **24h**  **48h** | **1.00 ± 0.33**  **0.80 ± 0.18**  **3.38 ± 0.83**  **1.45 ± 0.49** | **9.37 ± 2.56 (****)**  **0.79 ± 0.30 (ns)**  **3.82 ± 1.18 (ns)**  **0.39 ± 0.13 (****)** | **2.13 ± 0.59 (****)**  **0.97 ± 0.23 (*)**  **2.89 ± 0.85**  **0.28 ± 0.15 (****)** |

| **TIM23** | | | |
| --- | --- | --- | --- |
|  | Non-infected | *Hp 26695* | *Hp 26695∆vacA* |
| **2h**  **6h**  **24h**  **48h** | **1.00 ± 0.14**  **0.56 ± 0.06**  **1.82 ± 0.16**  **1.63 ± 0.53** | **1.58 ± 0.21 (****)**  **0.49 ± 0.08 (**)**  **1.23 ± 0.23 (****)**  **1.09 ± 0.27 (***)** | **0.52 ± 0.04 (****)**  **0.44 ± 0.05 (****)**  **1.51 ± 0.21 (****)**  **0.58 ± 0.17 (****)** |

**Table S2. Relative abundance of TOM22 and TIM23 signal normalized to the mitochondrial mass in AGS gastric epithelial cells infected with *Hp* 26695 and *Hp 26695∆vacA*.** Values indicate the ratio of immunofluorescence intensity of TOM22 or TIM23 over MitoTracker fluorescence signal (± SD). The value of non-infected cells at 2h was established as 1, and the other values were calculated accordingly. The ratios were calculated using the respective mean/mean values for 30 cells per sample from three independent experiments. Two-tailed Welch’s t test for each condition (n=3), infected *vs* non-infected at the same time-point; p< 0.05*; p< 0.001**; p< 0.001***; p< 0.0001****; ns: not significant. Significance was confirmed also with the Mann-Whitney test (n=3), not shown.

**A)**

| **Percentage of intramitochondrial POLG (POLG^+^/TOM22^+^)** | | | |
| --- | --- | --- | --- |
|  | Non-infected | *Hp 26695* | *Hp 26695∆vacA* |
| **2h**  **6h**  **24h**  **48h** | **93.38** ± 1.01  **89.56** ± 0.85  **92.73** ± 2.14  **93.43** ± 1.68 | **61.92** ± 2.5  **68.74** ± 5.41  **53.84** ± 5.49  **68.05** ± 6.18 | **50.82** ± 5.68  **75.30** ± 2.99  **60.83** ± 6.87  **64.28** ± 4.91 |

| **Percentage of mitochondria displaying POLG signal (TOM22^+^/POLG^+^)** | | | |
| --- | --- | --- | --- |
|  | Non-infected | *Hp 26695* | *Hp 26695∆vacA* |
| **2h**  **6h**  **24h**  **48h** | **89.62** ± 1.30  **88.72** ± 0.85  **95.35** ± 0.89  **89.55** ± 1.24 | **99.90** ± 4.26  **82.58** ± 1.76  **51.87** ± 7.23  **76.91** ± 6.60 | **6.42** ± 0.87  **95.22** ± 1.79  **66.33** ± 3.51  **62.32** ± 4.34 |

**B)**

| **Percentage of intramitochondrial TFAM (TFAM^+^/TOM22^+^)** | | | |
| --- | --- | --- | --- |
|  | Non-infected | *Hp 26695* | *Hp 26695∆vacA* |
| **2h**  **6h**  **24h**  **48h** | **91.90** ± 4.79  **93.16** ± 1.36  **89.82** ± 1.70  **94.44** ± 1.51 | **83.14** ± 2.34  **80.98** ± 4.09  **79.79** ± 5.51  **73.02** ± 2.07 | **71.30** ± 6.62  **83.84** ± 4.38  **89.82** ± 2.85  **77.36** ± 2.73 |

| **Percentage of mitochondria displaying TFAM signal (TOM22^+^/TFAM^+^)** | | | |
| --- | --- | --- | --- |
|  | Non-infected | *Hp 26695* | *Hp 26695∆vacA* |
| **2h**  **6h**  **24h**  **48h** | **88.82** ± 3.80  **91.30** ± 0.80  **94.04** ± 1.35  **84.55** ± 1.78 | **99.80** ± 0.03  **72.44** ± 2.21  **62.97** ± 7.00  **80.98** ± 3.07 | **19.00** ± 1.43  **64.18** ± 3.09  **88.58** ± 3.39  **87.06** ± 1.88 |

**Table S3: Effect of *Hp* infection on the mitochondrial localization of POLG and TFAM in gastric epithelial cells (AGS).** Values correspond to the percentage of **A)** POLG and **B)** TFAM localized in mitochondria (upper table, POLG^+^/TOM22^+^ and TFAM^+^/TOM22^+^) and of mitochondria with POLG and TFAM staining (lower table, TOM22^+^/POLG^+^ and TOM22^+^/TFAM^+^). Co-localization values are calculated for 30 cells/condition from three independent experiments; mean ±SEM. Note that at 2h pi with *Hp*26695 or *Hp*26695∆*vacA* only 61.9±2.5% or 50.8±5.7%, respectively, of POLG signal overlapped with mitochondria (POLG^+^/TOM22^+^ colocalization; see also Fig. 4A merge), indicating that at least one third of POLG remained extra-mitochondrial upon infection, and this event was largely independent of VacA. However, VacA was necessary for the distribution of POLG in mitochondria, as the fraction of mitochondria with POLG (TOM22^+^/POLG^+^, see also Figure 4A merge) accounted for only 6.4±0.9% in *Hp*26695∆*vacA-*infected cells *vs* 99.9±4% with *Hp*26695. In the case of TFAM the fraction of mitochondria with TFAM (TOM22^+^/TFAM^+^, see also Fig. 4D merge) accounted for 19±1.43% in the absence of VacA. In non-infected cells, under all conditions, POLG and TFAM were located in TOM22^+^ regions, as expected.

**A)**

| **Percentage of intramitochondrial POLG (POLG^+^/TOM22^+^)** | | | | |  | |
| --- | --- | --- | --- | --- | --- | --- |
|  | Untreated | Mock | VacA(wt) | VacA-(∆6-27) | |  |
| **2h**  **6h**  **24h**  **48h** | **93.38** ± 1.01  **89.56** ± 0.85  **92.73** ± 2.14  **93.43** ± 1.68 | **92.25** ± 3.54  **88.78** ± 0.95  **91.57** ± 1.85  **93.58** ± 2.24 | **76.42** ± 2.71  **37.30** ± 9.25  **89.06** ± 0.61  **52.20** ± 2.09 | **54.52** ± 2.20  **80.34** ± 2.14  **74.86** ± 5.42  **61.50** ± 2.94 | |  |

| **Percentage of mitochondria displaying POLG signal (TOM22^+^/POLG^+^)** | | | | |  | |
| --- | --- | --- | --- | --- | --- | --- |
|  | Untreated | Mock | VacA(wt) | VacA-(∆6-27) | |  |
| **2h**  **6h**  **24h**  **48h** | **89.62** ± 1.30  **88.72** ± 2.07  **95.35** ± 0.89  **89.55** ± 1.24 | **87.54** ± 3.25  **89.58** ± 1.45  **91.68** ± 1.45  **88.57** ± 1.58 | **33.06** ± 2.89  **1.20** ± 0.24  **99.84** ± 0.04  **16.34** ± 1.30 | **28.18** ± 1.47  **98.82** ± 0.24  **2.90** ± 0.05  **99.94** ± 0.02 | |  |

**B)**

| **Percentage of intramitochondrial TFAM (TFAM^+^/TOM22^+^)** | | | |  |
| --- | --- | --- | --- | --- |
|  | Untreated | Mock | VacA(wt) | VacA-(∆6-27) |
| **2h**  **6h**  **24h**  **48h** | **91.90** ± 4.79  **93.16** ± 1.36  **89.82** ± 1.70  **94.44** ± 1.51 | **91.91** ± 0.78  **94.50** ± 1.15  **90.25** ± 1.25  **93.80** ± 1.58 | **58.08** ± 2.91  **96.60** ± 2.02  **96.56** ± 1.51  **93.56** ± 1.58 | **85.78** ± 1.46  **94.14** ± 1.30  **97.70** ± 0.50  **98.20** ± 0.56 |

| **Percentage of mitochondria displaying TFAM signal (TOM22^+^/TFAM^+^)** | | | | |  | |
| --- | --- | --- | --- | --- | --- | --- |
|  | Untreated | Mock | VacA(wt) | VacA-(∆6-27) | |  |
| **2h**  **6h**  **24h**  **48h** | **88.82** ± 3.80  **91.30** ± 0.80  **94.04** ± 1.35  **84.55** ± 1.78 | **87.89** ± 3.58  **91.50** ± 1.87  **92.50** ± 1.58  **85.87** ± 0.75 | **98.88** ± 0.01  **45.04** ± 6.07  **12.02** ± 3.15  **45.56** ± 4.64 | **99.20** ± 0.24  **41.12** ± 2.63  **50.72** ± 1.39  **24.70** ± 2.48 | |  |

**Table S4: Effect of VacA(wt) and VacA-(∆6-27) proteins on the mitochondrial localization of POLG and TFAM in gastric epithelial cells (AGS).** Values correspond to the percentage of **A)** POLG and **B)** TFAM localized in mitochondria (upper table, POLG^+^/TOM22^+^ and TFAM^+^/TOM22^+^) and of mitochondria with POLG and TFAM staining (lower table, TOM22^+^/POLG^+^ and TOM22^+^/TFAM^+^). Co-localization values are calculated for 30 cells/condition from three independent experiments; mean ± SEM. VacA may affect the ability of POLG either to reach or be imported into mitochondria, since only 33± 2.9% and 28%±2 of mitochondria (TOM22^+^) were POLG^+^ after 2h of treatment with VacA(wt) or VacA-(∆6-27), respectively (lower panel A). In the case of TFAM 99% of mitochondria were TFAM^+^ at 2h pi in cells either treated with VacA(wt) or VacA-(∆6-27). Untreated: AGS cells; Mock: AGS cells incubated with the VacA activation buffer as control.


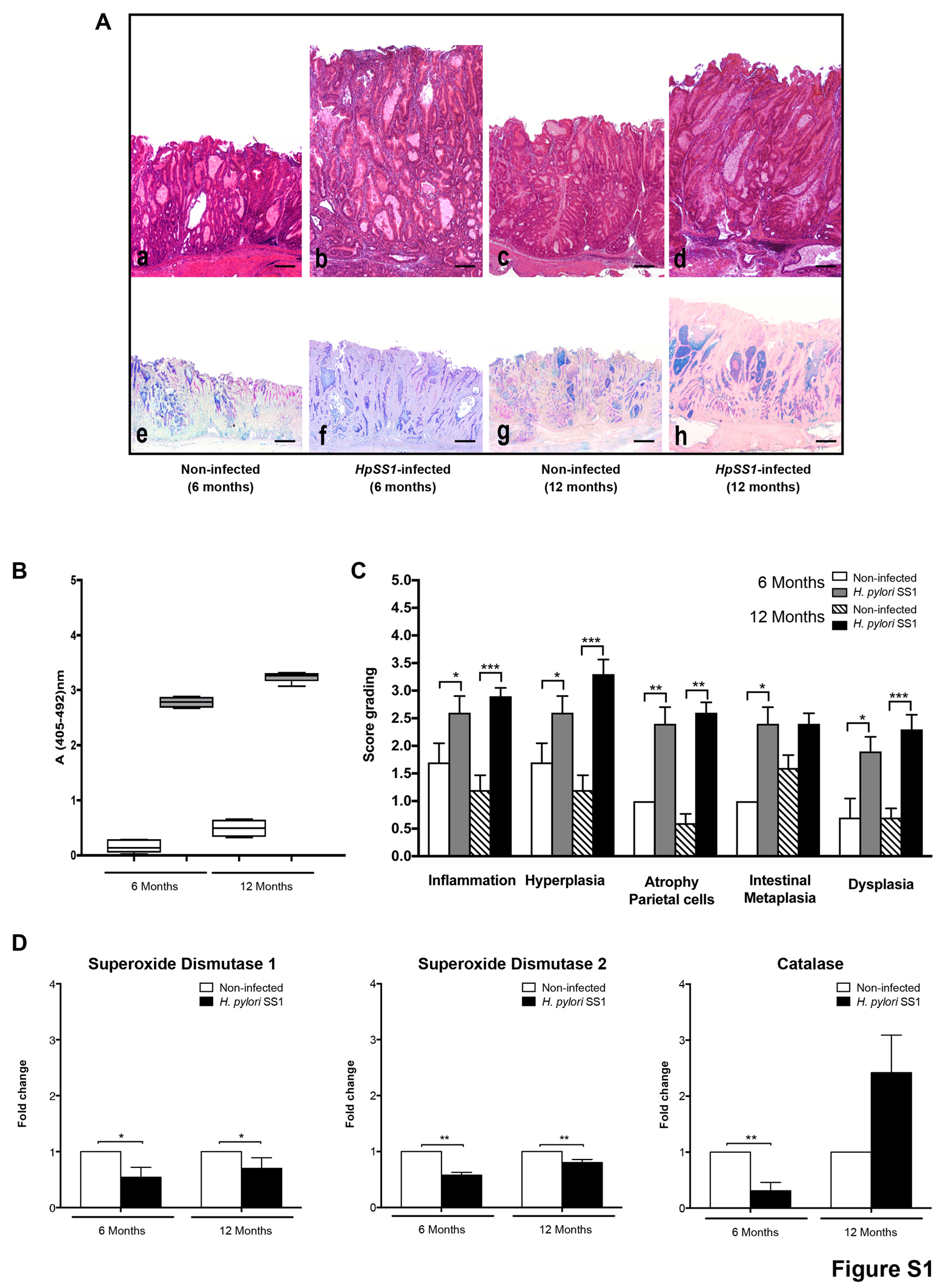


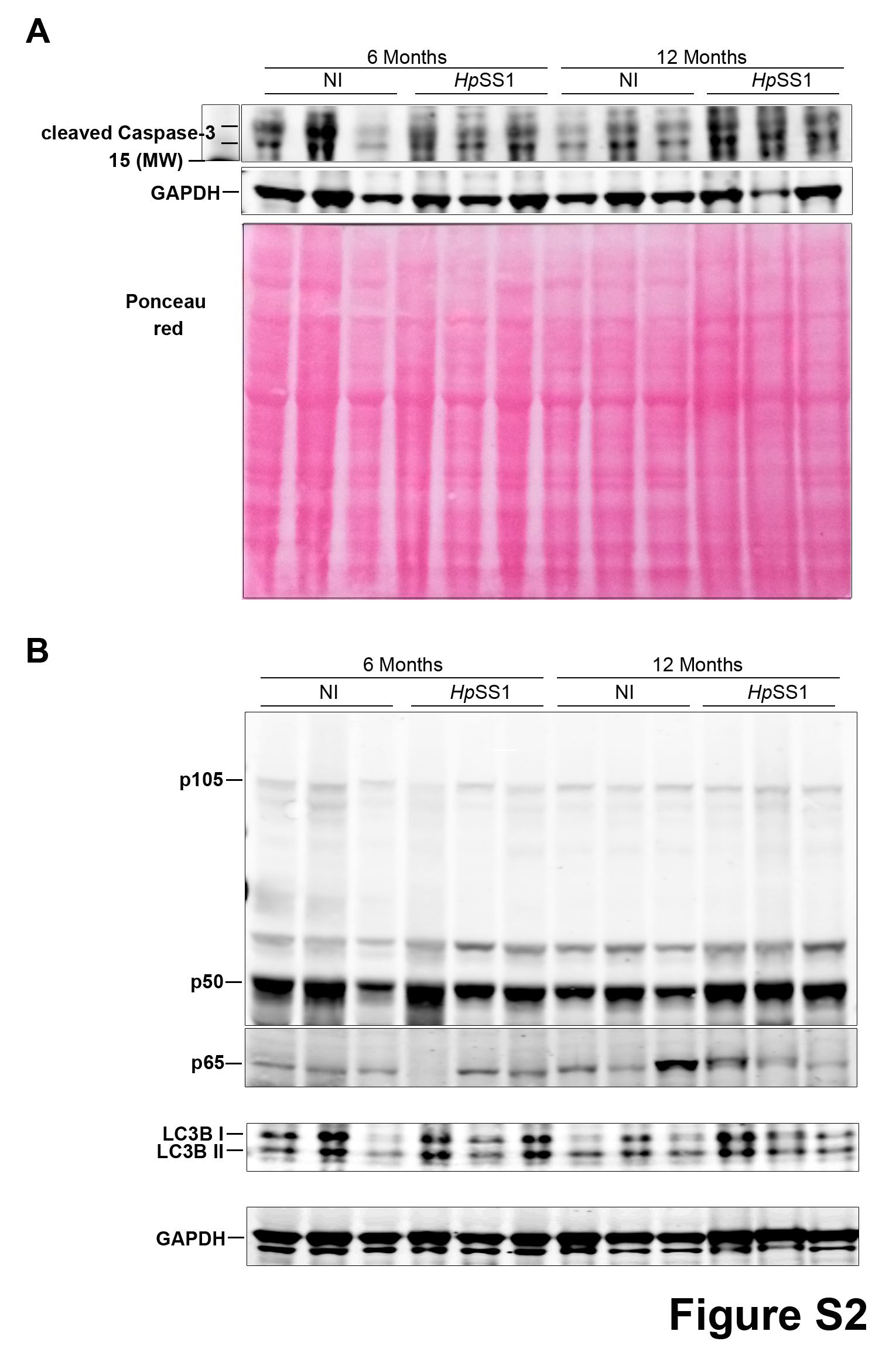


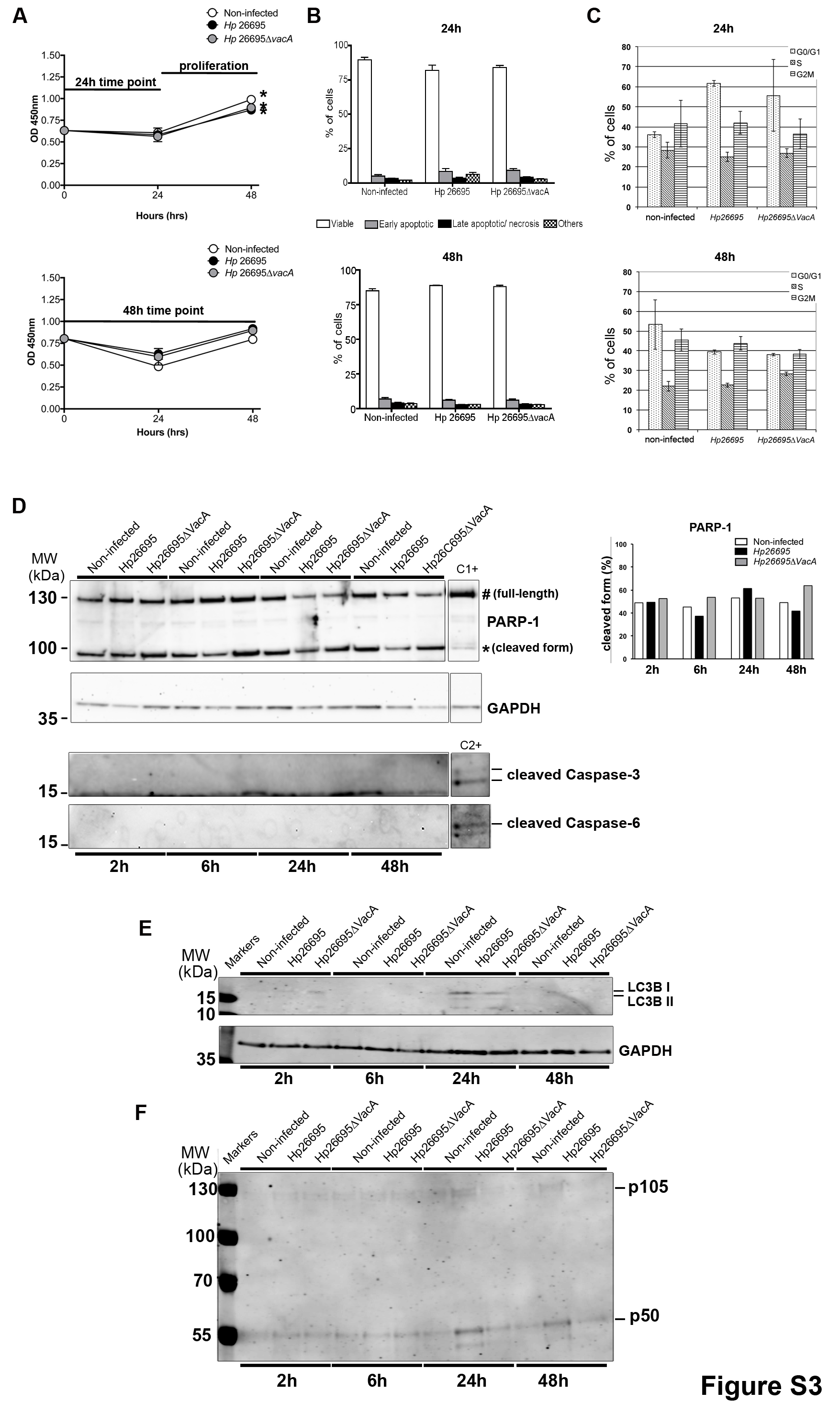

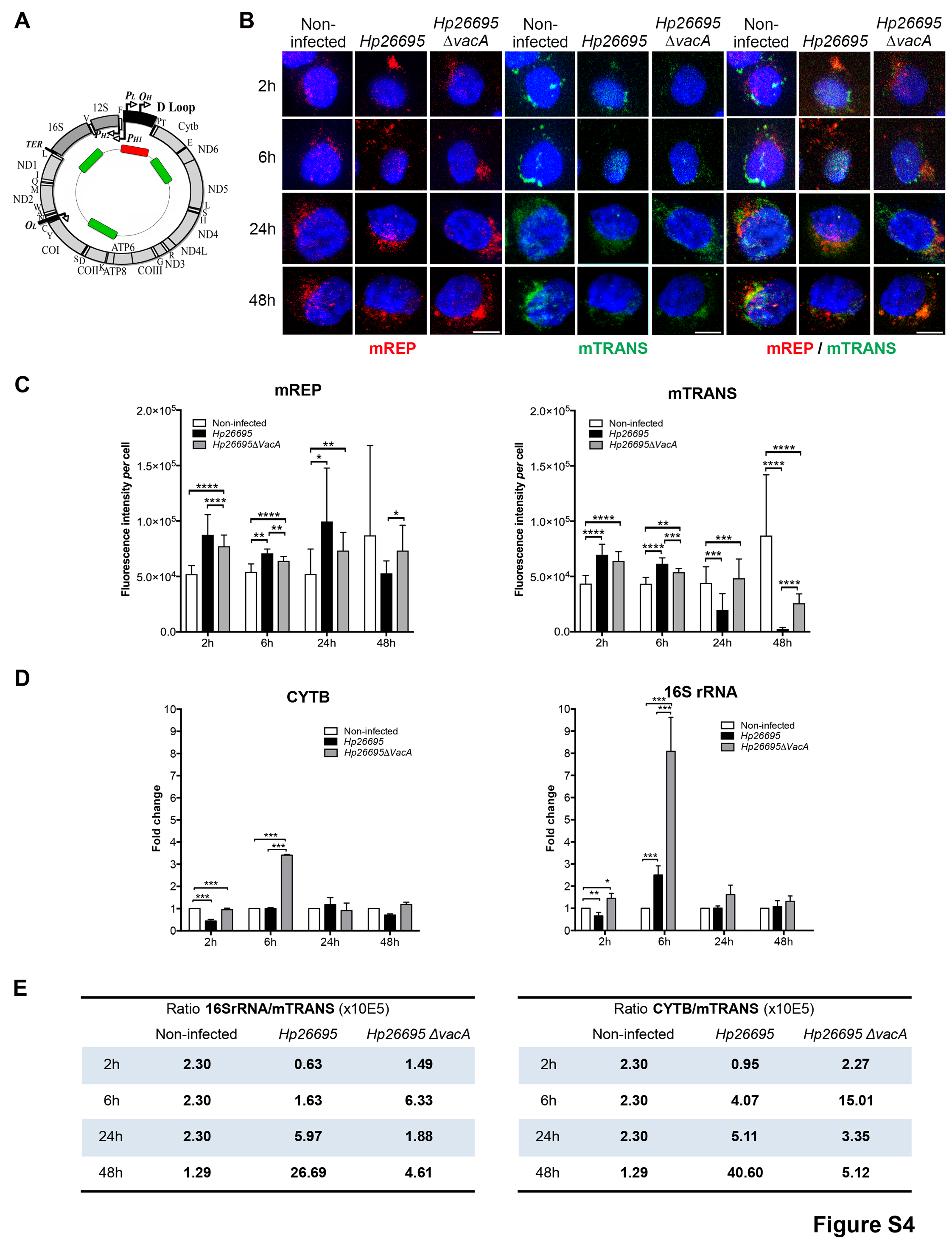


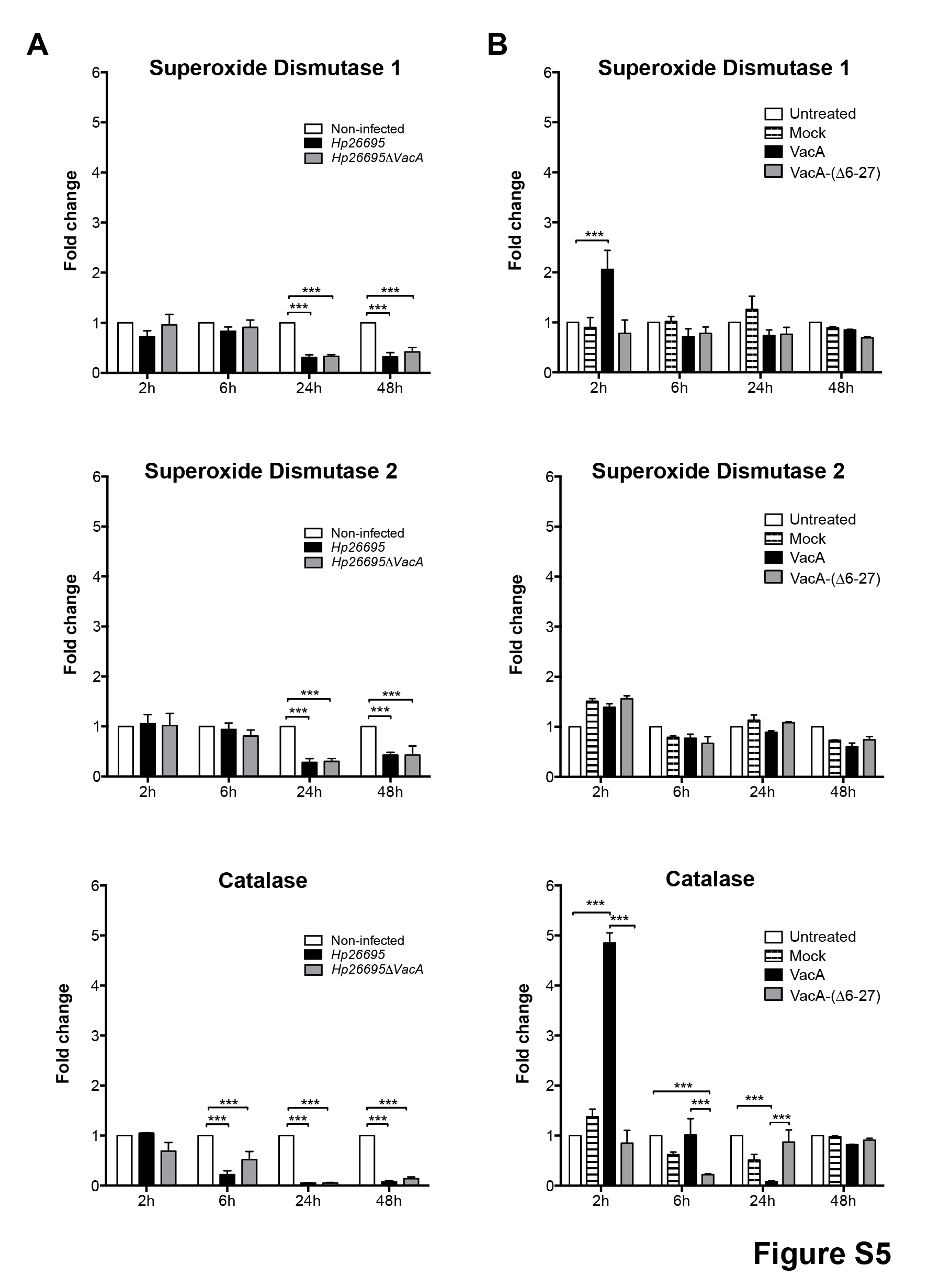


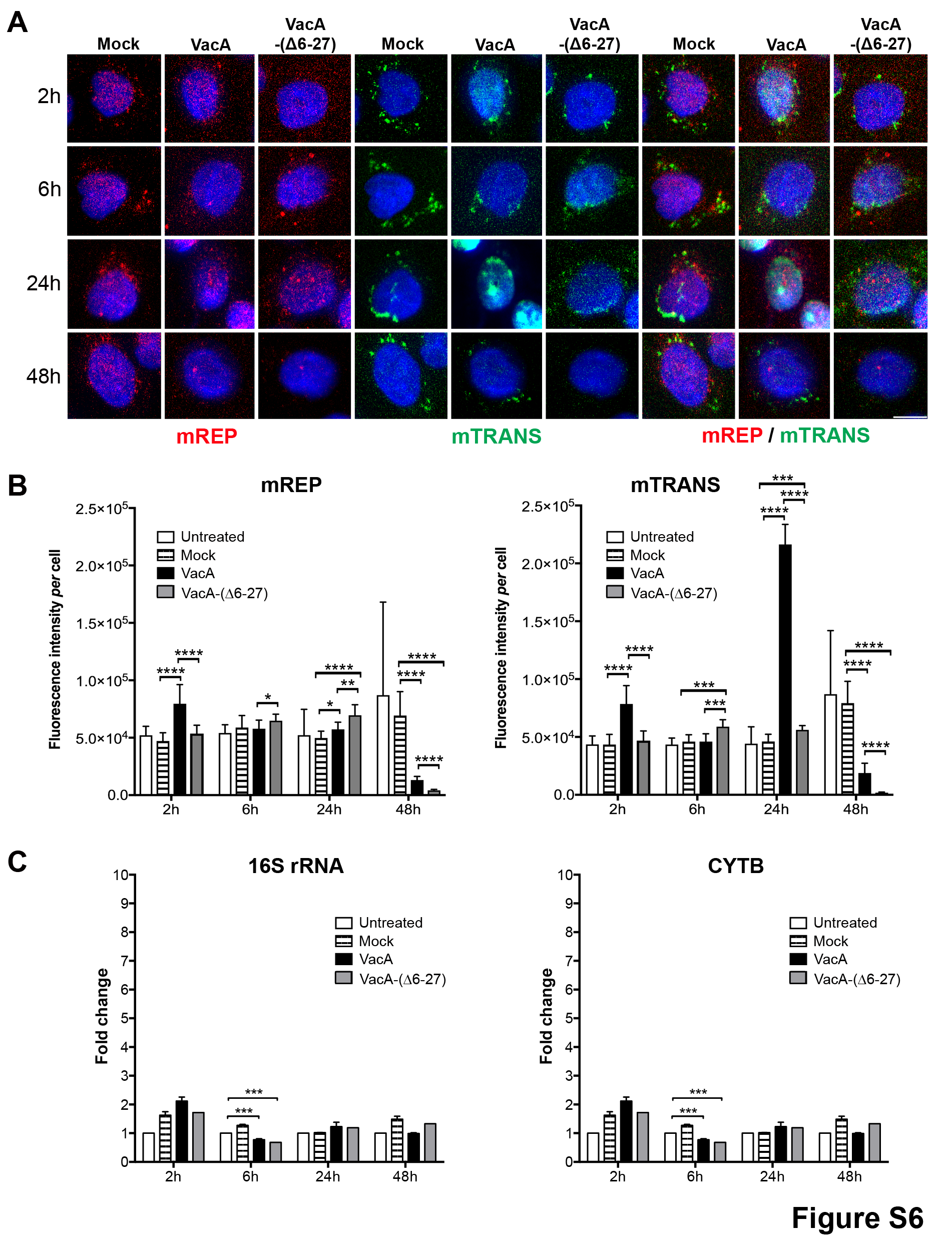


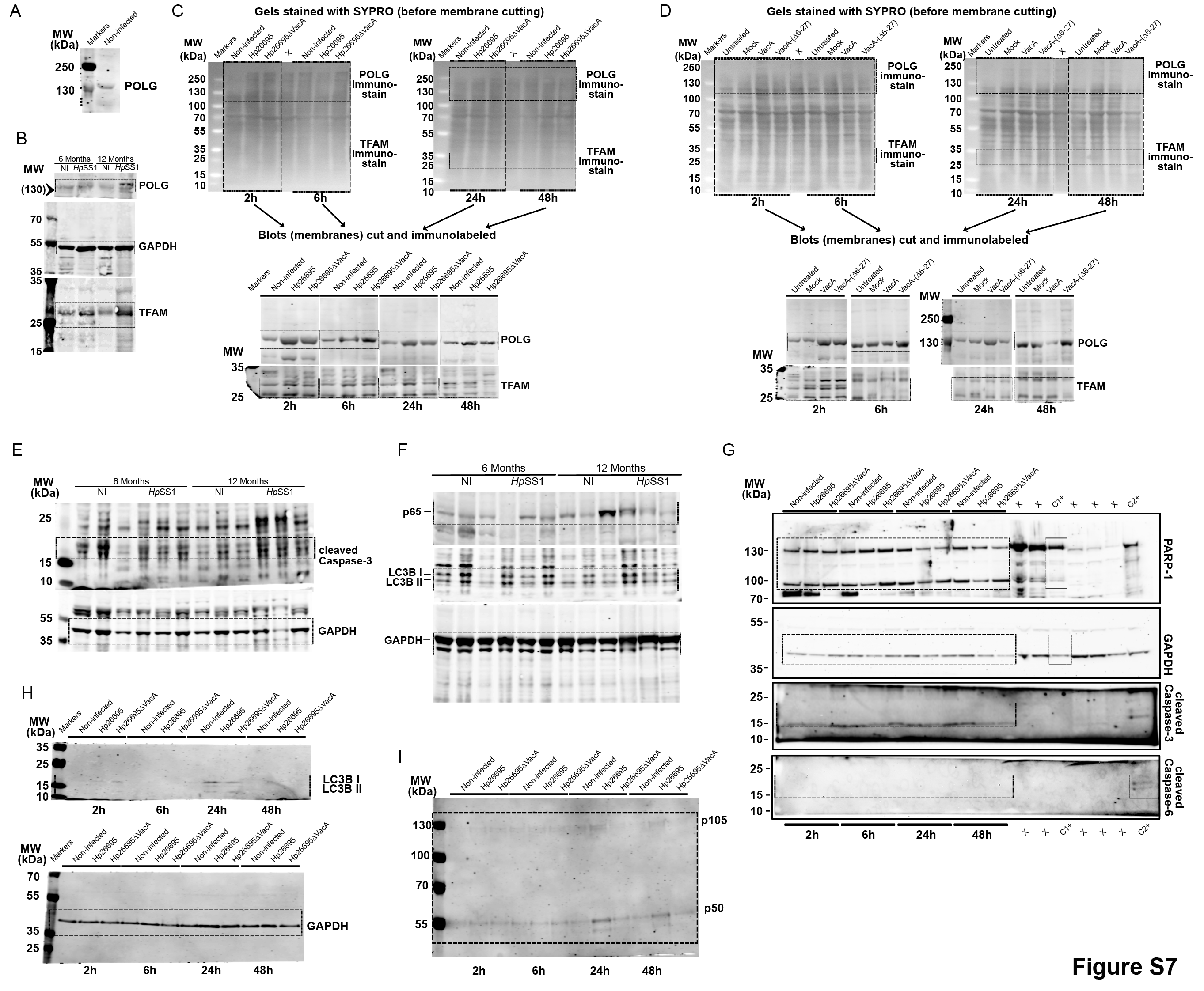

Supplement: Supplementary file 1 — Supplementary Information [file 41598_2017_15567_MOESM1_ESM.docx]
